# Synthesis and properties of fully-conjugated indacenedithiophenes†

**DOI:** 10.1039/c3sc53181c

**Published:** 2013-12-10

**Authors:** Brian S. Young, Daniel T. Chase, Jonathan L. Marshall, Chris L. Vonnegut, Lev N. Zakharov, Michael M. Haley

**Affiliations:** a Department of Chemistry & Biochemistry and Materials Science Institute, University of Oregon Eugene OR 97403-1253 USA haley@uoregon.edu +1 541-346-0487 +1 541-346-0456

## Abstract

The synthesis and characterization of four fully-conjugated indacenedithiophenes (IDTs) are disclosed. In contrast to anthradithiophenes, regioselective synthesis of both *syn* and *anti* isomers is readily achieved. Thiophene fusion imparts increased paratropic character on the central indacene core as predicted by DFT calculations and confirmed by ^1^H NMR spectroscopy. IDTs exhibit red-shifted absorbance maxima with respect to their all-carbon analogues and undergo two-electron reduction and one-electron oxidation.

## Abstract 2

The regioselective synthesis and optoelectronic characterization of four fully-conjugated, isomeric indacenedithiophenes are disclosed.

## Graphical abstract

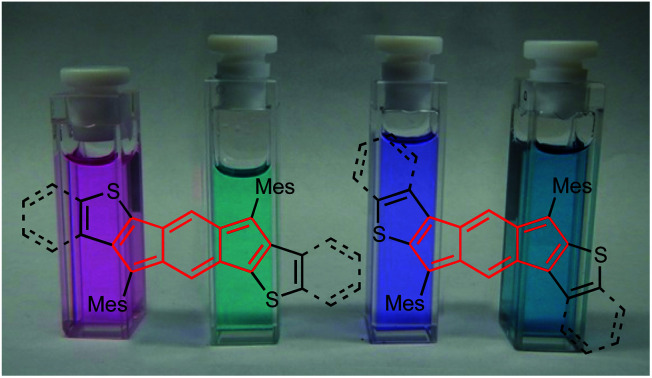

## Introduction

**Fig. 1:**
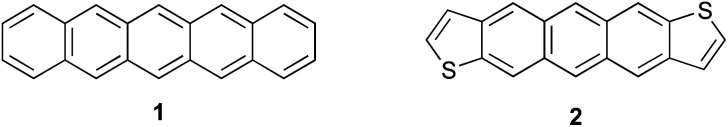
Pentacene (1) and structural analogue anthradithiophene (ADT, 2).

In recent years, there has been tremendous interest in highly conjugated polycyclic hydrocarbons, such as the higher acenes, because of their fascinating optical and electronic properties.^1^ Although pentacene (1, Fig. 1) and its derivatives have been utilized in device applications such as field effect transistors, photovoltaics, and light emitting diodes, these molecules are susceptible to oxidative and photolytic degradation;^2^ therefore, alternative, acene-like topologies have been explored.^3–6^ One of the initial substitutes for pentacene was structurally analogous anthradithiophene (ADT, 2), as inclusion of heterocycles allows for tuning of physical and electronic properties.^7^ Thieno-fusion as part of the acene skeleton is a particularly attractive option for a number of reasons including high electron mobilities, increased stability, and ease of functionalization. In fact, hole mobilities of 2 and derivatives approach values observed for 1 yet the former exhibit improved oxidative stability.^8^ As a whole, these structurally-related acene molecules and myriad derivatives behave typically as organic p-type compounds. Comparatively, there are considerably fewer organic n-type structures in the literature; thus, there is a pressing need for new molecular frameworks that can transport electrons.

**Fig. 2:**
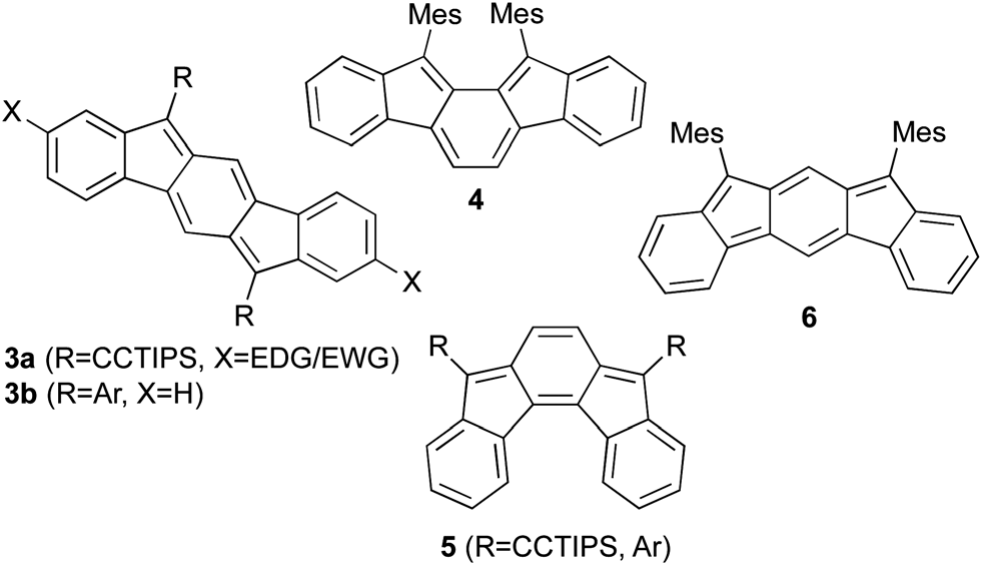
Recently reported indenofluorene structures 3–6.

Very recently, our group^9^ and others^10^ have begun to examine the isomers of indenofluorenes^11^ (IFs, Fig. 2) as potential n-type materials due to their ability to reversibly accept two electrons. The stability and electronic properties of the IFs can be tuned by functionalization at a number of positions. Our initial studies showed that a range of electron-rich and electron-poor groups at the 2 and 8 positions of the indeno[1,2-*b*]fluorene skeleton (*e.g.*, 3a)^9*b*^ had only a modest effect on the electronic properties of these compounds; however, we demonstrated subsequently that functionalizing the [1,2-*b*]IFs with aryl groups at the 6 and 12 positions (3b)^9*c*^ resulted in much greater variability in the redox properties of the molecules, with some displaying amphoteric behaviour. Tobe and our group reported, respectively, that [2,1-*a*] isomer 4^10*a*^ and previously unknown [2,1-*c*] isomer 5^9*d*^ also possessed excellent electrochemical properties with smaller HOMO-LUMO gaps than the [1,2-*b*]IFs. Tobe *et al.* very recently described unknown [2,1-*b*] isomer 6;^10*c*^ however, the instability of the molecule may preclude its use in organic electronics.

**Fig. 3:**
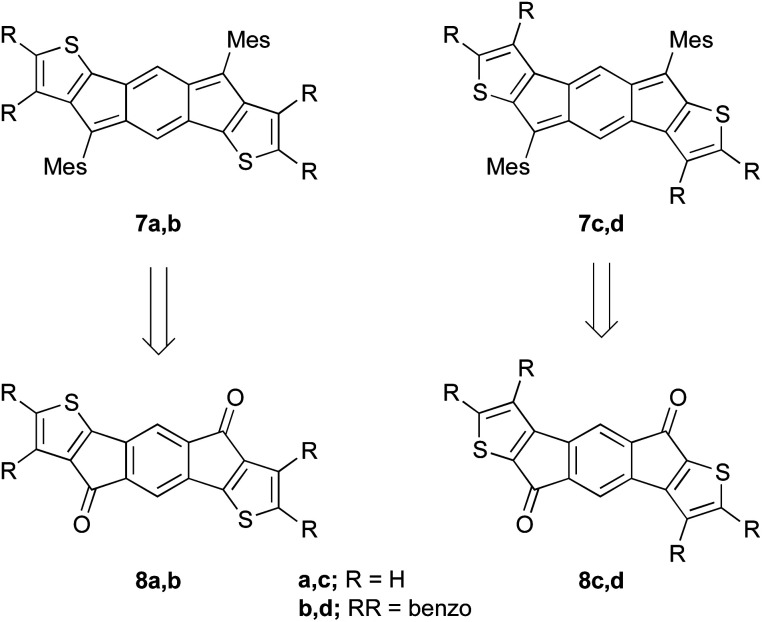
Targeted structures of IDTs 7a,c and IDBTs 7b,d synthesized from the corresponding diones 8a–d.

Given the analogy between pentacene and anthradithiophene, we were eager to build upon our previous successes and thus examine a similar structural analogy between the indeno[1,2-*b*]fluorene and indacenodithiophene skeletons. Herein we report the synthesis of two indacenedithiophenes (IDTs, 7a,c) and two indacenedibenzothiophenes (IDBTs, 7b,d) from the corresponding indacenedione precursors (8a–d, Fig. 3), along with the respective optical, electrochemical, computational, and structural data for this new class of electron-accepting molecules.

## Results and discussion

**Fig. 4:**
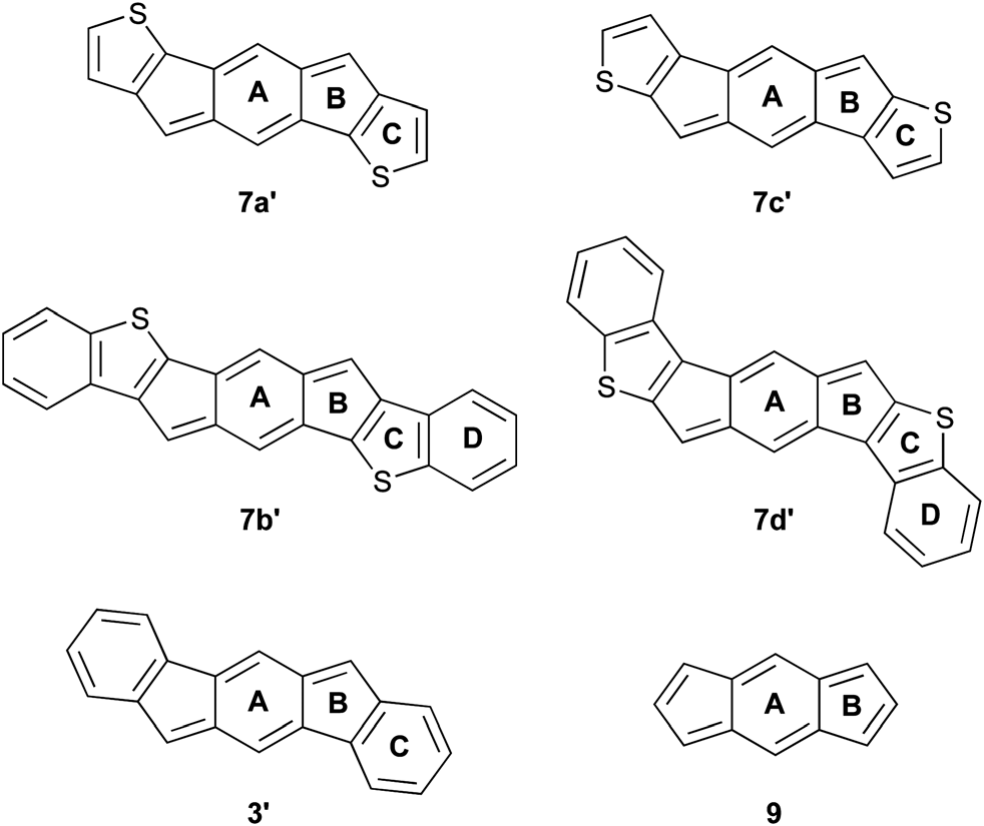
Model IDTs/IDBTs 7a′–d′, [1,2-*b*]IF 3′ and *s*-indacene 9 used for NICS(1) calculations.

Our initial studies *in silico* of the simplified IDT structures 7a′–d′ (Fig. 4) suggested that the 20 π-electron, formally antiaromatic compounds should possess some interesting optical and electronic properties. NICS(1) calculations (Table 1) indicated that, compared to model [1,2-*b*]IF 3′, the weaker aromaticity of the fused thiophenes (ring C in 7a′–d′) would allow the antiaromaticity of the indacene core (rings A and B) to reassert itself to *ca.* 60–90% of that found for *s*-indacene (9). Calculations predicted lower low-energy transitions in the absorption spectrum, similar to what is observed with other strongly paratropic molecules.^12^ The DFT calculations also predicted lower HOMO/LUMO energy levels and smaller energy gaps than for structurally analogous IFs. If these predictions hold true, then IDTs will differ significantly from ADTs, which show an *increase* in their gap energies compared to analogously derivatized pentacenes.^13^

**Table 1:** Calculated NICS(1)^a^ values for rings A–D of IDTs 7a′–d′, [1,2-*b*]IF 3′ and *s*-indacene 9

Entry	A	B	C	D
3′	2.42	3.18	−6.80	na
7a′	7.89	11.01	−4.55	na
7b′	9.51	13.54	−4.25	−9.09
7c′	7.79	10.72	−4.37	na
7d′	12.04	16.11	−4.93	−8.37
9	12.91	15.41	na	na

a DFT (B3LYP/6-311G**).

**Scheme 1:**
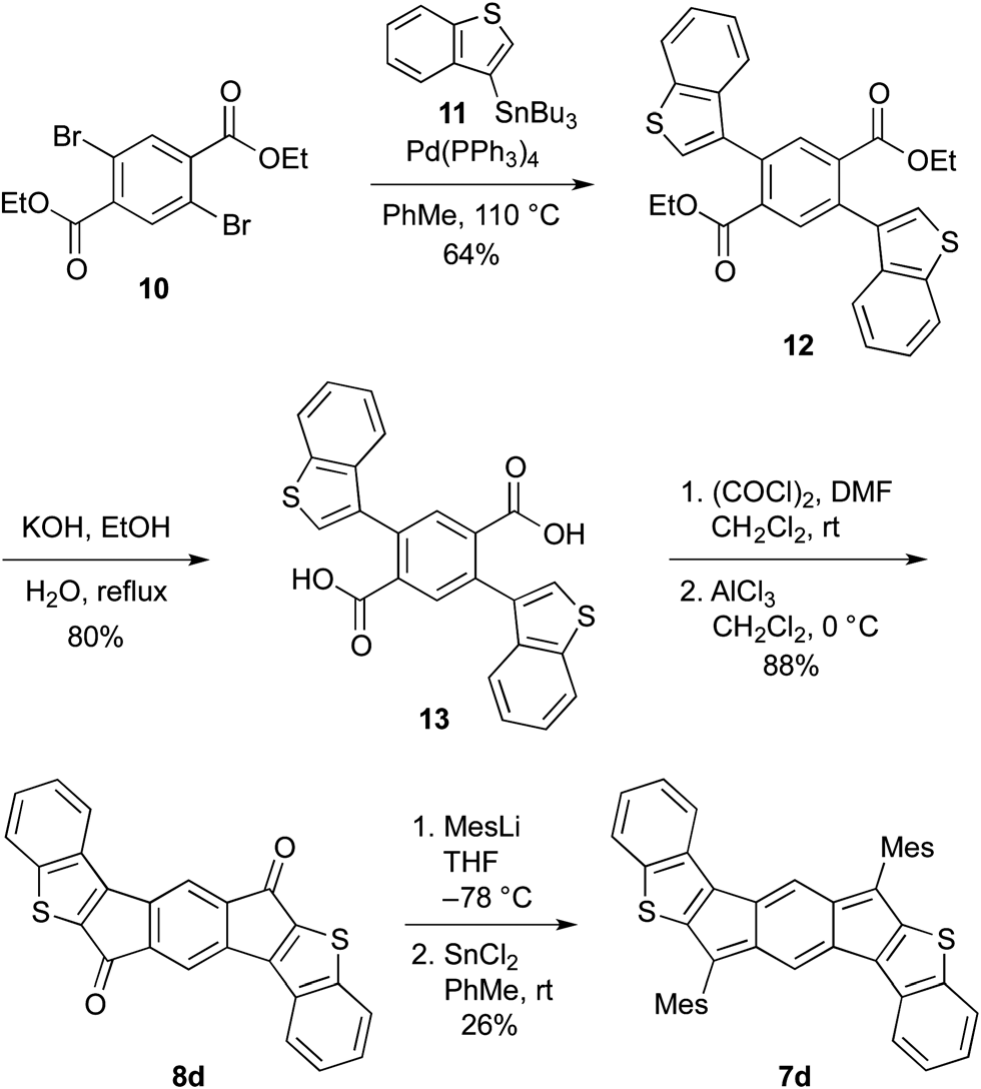
Synthesis of IDBT 7d.

The preparation of indacenedithiophenes 7a,c and indacene-dibenzothiophenes 7b,d followed the typical pathway to generate indenofluorenes—addition of a nucleophile to indacenediones 8a–d followed by SnCl_2_-mediated dearomatization. We elected to use mesityl lithium, anticipating based on the calculations that the bulky group would be needed to help kinetically stabilize the indacene core. Of the requisite diones, only 8a is known;^14^ diones 8b–d were produced using a similar synthetic strategy, which is modified from the procedure used by McCulloch *et al.*^14*a*^ Shown for 7d/8d in Scheme 1, cross-coupling dibromide 10^15^ to stannane 11^16^ under Stille conditions generated diester 12, which was subsequently saponified to diacid 13. Conversion to the acid chloride followed by intramolecular Friedel–Crafts acylation furnished dione 8d. Treatment with MesLi gave the crude diol, which in turn was reductively dearomatized with SnCl_2_, affording fully conjugated 7d (see ESI† for the preparation of all other compounds). A distinct advantage to this synthetic route is that the IDTs possess a defined structure, whereas ADT and its derivatives have typically been prepared and studied as an inseparable mixture of *syn*- and *anti*-isomers as a result of the regiorandom Aldol condensations used to generate the precursor dione molecules.^13,17^

IDTs 7a,c and IDBTs 7b,d were isolated as stable, dark blue-green solids in modest overall yields. As anticipated from the calculations, the ^1^H NMR spectra of 7a–d corroborate the stronger antiaromatic character of the IDTs: the signal for the protons on ring A appear at *ca.* 6.1 ppm, whereas the same protons in diones 8a–d appear at about 7.3 ppm, and at about 7.1 ppm for the ring A protons in derivatives of 3 and 4.

**Fig. 5:**
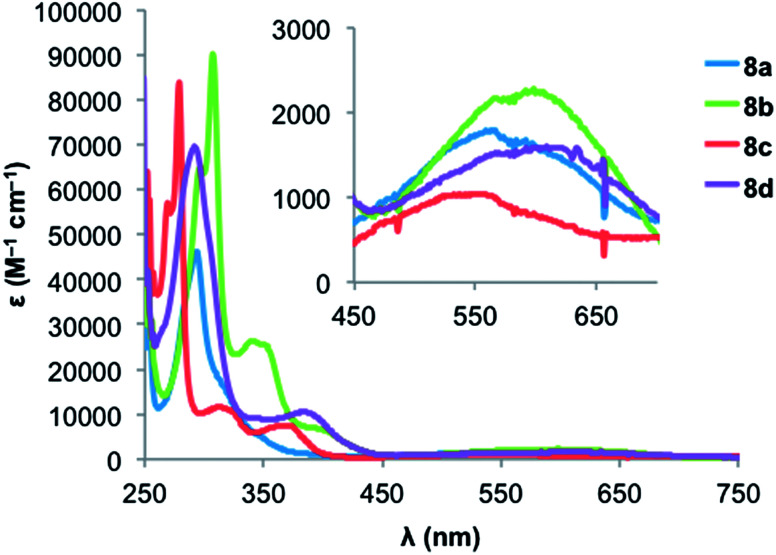
Electronic absorption spectra of diones 8a–d in DMSO.

**Fig. 6:**
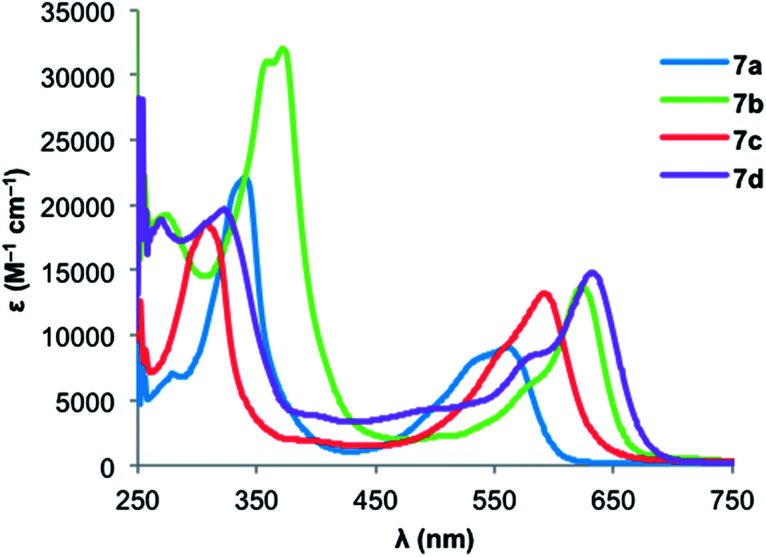
Electronic absorption spectra of IDTs 7a–d in DMSO.

Fig. 5 and 6 show the electronic absorption spectra for diones 8 and IDTs 7, respectively. These data along with calculated and experimental HOMO and LUMO energies and energy gaps are summarized in Table 2. The spectra of diones 8 display intense absorptions from approximately 275 nm to 325 nm with broad absorption bands attributable to weak π → π* transitions appearing in the 550–600 nm range. The IDTs show maximum absorbance peaks ranging from 315 to 375 nm, but it is the lower energy absorptions that reveal clear differences between the thiophene-containing structures: (1) not surprisingly, the extended conjugation in IDBTs 7b,d results in a lower *λ*_max_ (624/632 nm) compared to the analogous IDTs 7a,c (561/592 nm). (2) The “*syn*” isomers 7c,d (S atom and Mes of adjacent rings on same side) possess a lower *λ*_max_ compared to the analogous “*anti*” isomers 7a,b (S atom and Mes of adjacent rings on opposite side). (3) As predicted by the calculations, the IDTs show a *decrease* in their gap energies by 0.2–0.3 eV compared to the analogous dimesityl derivative of indenofluorene 3b (low energy *λ*_max_ of 516 nm),^9*c*^ which is in marked contrast to the aforementioned pentacene/ADT comparison.^13^

**Table 2:** Computational, electrochemical, and optical data for IDTs 7a–d and diones 8a–d

Compd	Computational^a^			Electrochemical^b^							Optical^c^
	*E*_HOMO_	*E*_LUMO_	*E*_gap_	*E* ^1^_red_	*E* ^2^_red_	*E*_ox_	*E*_HOMO_	*E*_LUMO_	*E*_gap_	*λ*_max_	*E*_gap_
7a	−5.35	−3.17	2.18	−0.92	−1.69^d^	0.93	−5.57	−3.72	1.85	561	2.07
7b	−5.30	−3.34	1.96	−0.80	−1.62^d^	0.92	−5.56	−3.84	1.72	624	1.88
7c	−5.41	−3.22	2.18	−0.94	−1.59^d^	0.93^d^	−5.57^d^	−3.70	1.88	592	1.96
7d	−5.29	−3.46	1.84	−0.61	−1.24^d^	0.98^d^	−5.62^d^	−4.03	1.59	632	1.83
8a^e^	−6.12	−3.40	2.72	−0.91	−1.49	—	—	−3.73	—	566	1.75
8b^f^	−5.99	−3.47	2.51	—	—	—	—	—	—	598	1.66
8c^e^	−6.22	−3.41	2.81	−0.87	−1.27	—	—	−3.77	—	551	1.89
8d^f^	−5.94	−3.51	2.43	—	—	—	—	—	—	609	1.56

a Calculations were performed at the B3LYP/6-31G** level of theory; energies are in eV.

b CVs were recorded using 1–5 mM of analyte in 0.1 M Bu_4_NOTf/CH_2_Cl_2_ at a scan rate of 50 mV s^−1^ with a glassy carbon working electrode, a Pt coil counter electrode, and a Ag wire pseudo-reference. Values reported as the half-wave potential (*vs.* SCE) using the Fc/Fc^+^ couple (0.46 V) as an internal standard. HOMO and LUMO energy levels in eV were approximated using SCE = −4.68 eV *vs.* vacuum (see ref. 18) and *E*_1/2_ values for reversible processes or *E*_p_ values for irreversible processes.

c Spectra were obtained in DMSO; wavelengths are in nm. The optical HOMO/LUMO gap was determined as the intersection of the *x*-axis and a tangent line passing through the inflection point of the lowest-energy absorption; energies are in eV.

d Reported as *V* at peak current, not half-wave potential.

e Due to poor solubility in CH_2_Cl_2_*o*-dichlorobenzene (ODCB) was used as solvent for electrochemical measurements.

f Cyclic voltammetry measurements could not be obtained due to poor solubility of the compound.

**Fig. 7:**
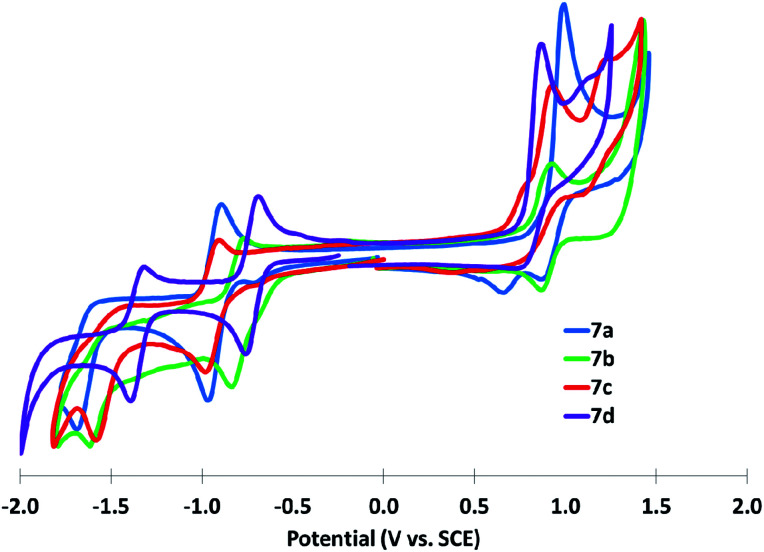
Cyclic voltammetry of IDTs 7a–d.

**Fig. 8:**
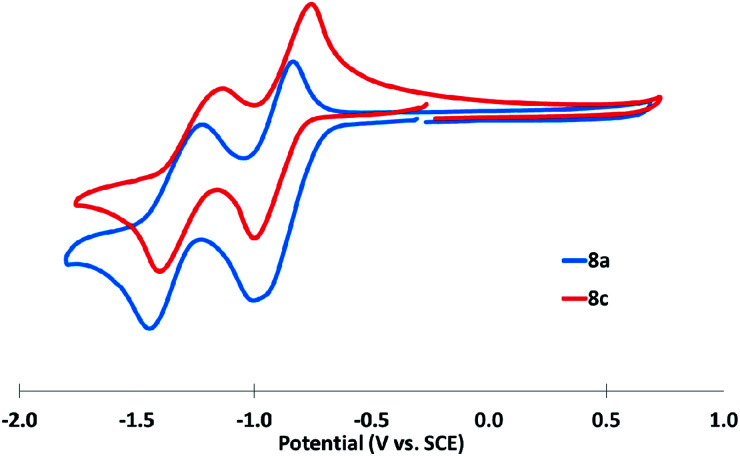
Cyclic voltammetry of diones 8a and 8c.

IDTs 7a,c and IDBTs 7b,d all undergo one reversible reduction in the solution state; however, the second reduction is essentially irreversible. The first oxidations of 7a and 7c were quasi-reversible and irreversible, respectively, whereas the first oxidations of 7b,d were fully reversible under the experimental conditions (Fig 7). The extended π conjugation of the IDBTs had no significant effect on the HOMO energy levels compared to the IDTs; however, IDBTs possess LUMO levels 0.15–0.25 eV lower in energy than the corresponding IDTs. Calculated HOMO levels are in good agreement with those measured by cyclic voltammetry (Fig. 7 and 8), though calculated LUMO levels are higher than experimental levels, which is common for DFT derived LUMO levels of molecules featuring the *p*-quinodimethane motif.^9^ Comparison of the HOMO and LUMO levels of similarly functionalized IFs shows that the HOMO is destabilized and the LUMO is stabilized by thiophene substitution compared to the all carbon analogues, resulting in a smaller bandgap as demonstrated by the longer wavelength *λ*_max_. The presence of two reductions in IFs is typically attributed to the stabilization of the dianion by aromatization of the formally antiaromatic indacene core to give a [4*n* + 2] π-electron system; similar behaviour was demonstrated for IDTs 7a,c and IDBTs 7b,d.

Diones 8a,c were similarly assessed *via* CV, and each displayed two reversible reductions, with no accessible oxidations under the experimental conditions (Fig. 8). The poor solubility of the corresponding dibenzodiones 8b,d in solvents amenable to electrochemical analysis precluded analogous investigation.

**Fig. 9:**
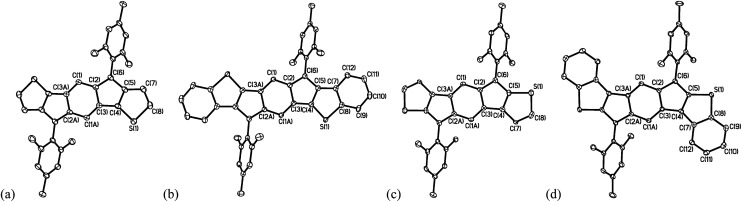
Molecular structures of (a) 7a, (b) 7b, (c) 7c and (d) 7d; hydrogen atoms omitted for clarity. Ellipsoids drawn at 50% probability level.

Crystals suitable for X-ray diffraction were grown by diffusion of acetonitrile into CH_2_Cl_2_ (7a,b,d) or by slow evaporation of CD_2_Cl_2_ (7c). The structures of all four C_2_-symmetric molecules are shown in Fig. 9; comparison of select bond lengths in the IDTs along with those in the dimesityl derivative of [1,2-*b*]IF 3b are given in Table 3.^19^ The lengths of the bonds in the central six-membered ring in 7a and 3b are quite similar (*Δ* 0.002–0.004 Å), while the bond lengths of the five-membered rings show more variability. This is not surprising given the five-membered rings are fused to thiophene rings in 7a, and benzene rings in 3b. Comparing 7a,c to their benzo-fused counterparts 7b,d, it can be seen that the bond lengths of the indacene core are more homogenous in 7b and 7d. This homogenization is indicative of increased paratropicity within this core in the benzo-fused IDTs, similar to what Hafner and co-workers observed for the 1,3,5,7-tetra-*tert*-butyl derivative of indacene 9,^20^ and is in agreement with NICS(1) values of rings A and B in Table 1. The dihedral angle between the average planes of the mesityl group and the IDT core is smaller for 7c (63.6°) and 7d (60.6°) than for 7a (68.2°) and 7b (74.3°), which presumably results in increased conjugation overall for the *syn* isomers compared to the *anti*. This could possibly explain the longer wavelength absorbances of the *syn* isomers compared to the *anti* isomers; otherwise, there are no significant structural differences between the *syn*/*anti* isomers.

**Table 3:** Select bond lengths (Å) of IDT 7a,c, IDBTs 7b,d and IF 3b

bond^a^	7a	7b	7c	7d	3b (R = Mes)^b^
C1–C2	1.431(2)	1.412(3)	1.418(3)	1.421(2)	1.433(3)
C1–C3A	1.360(2)	1.377(3)	1.363(3)	1.371(2)	1.356(2)
C2–C3	1.469(2)	1.457(3)	1.456(3)	1.454(2)	1.467(2)
C2–C6	1.388(2)	1.409(3)	1.398(3)	1.407(2)	1.380(2)
C3–C4	1.452(2)	1.437(3)	1.461(3)	1.457(2)	1.469(3)
C4–C5	1.389(2)	1.393(3)	1.384(3)	1.391(2)	1.413(2)
C5–C6	1.460(2)	1.435(3)	1.447(3)	1.441(2)	1.471(2)
C5–C7	1.417(2)	1.441(3)	1.425(3)^c^	1.429(2)^c^	na^e^
C7–C8	1.355(3)	1.418(3)	1.357(4)	1.423(2)	na^e^
C4–S1	1.706(2)	1.718(2)	1.720(2)^d^	1.730(2)^d^	na^e^
C8–S1	1.736(2)	1.756(2)	1.714(3)	1.749(2)	na^e^

a Numbering scheme shown in Fig. 9.

b See ref. 9c.

c C4–C7 in 7c,d.

d C5–S1 in 7c,d.

e Not applicable as the fused ring is benzene, not thiophene.

**Fig. 10:**
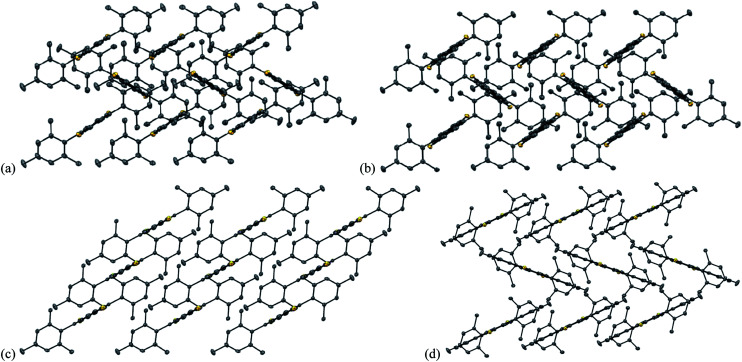
Packing diagrams of (a) 7a, (b) 7b, (c) 7c and (d) 7d; hydrogen atoms omitted for clarity.

Both 7a and 7b exhibit herringbone-like packing of the IDT core with the sulfur atoms participating in the closest intermolecular distances (Fig. 10). In the crystal structure of 7a, each sulfur atom makes two short contacts with the five-membered ring of the adjacent molecules (3.312 and 3.421 Å) with a relatively long S⋯S contact (4.202 Å). While 7b exhibits a similar crystal packing pattern, the extra benzo groups on this molecule force the sulfur atoms to be closest to the thiophene rings of the adjacent molecules; these S⋯C contacts are in the range 3.406–3.520 Å with the S⋯S contact of 3.707 Å.

The packing of 7d is slightly different than 7a and 7b but also herringbone-like. The shortest C⋯C contacts between the central ring of one molecule and the peripheral ring of the other are 3.499 and 3.352 Å; unfortunately, the closest contact is at a site with no significant LUMO density. IDT 7c also exhibits a 1D structure with slight overlap of the thiophene units in neighbouring molecules, with a distance between the average planes of 3.615 Å; however, the parallel arrangement of the 1D columns relative to each other in the packing of 7c is clearly different than the herringbone pattern in 7a, 7b and 7d (Fig. 10). The shortest S⋯S contact in 7c is 4.829 Å, showing that such interactions are not involved in directing the crystal packing.

## Conclusions

In summary, we have demonstrated the feasibility of fully conjugated indacenedithiophenes where both *syn* and *anti* isomers can be regioselectively synthesized. In tandem with computational findings, both optical and electrochemical data reveal stabilized HOMO and LUMO energy levels for 7a–d. Unlike the anthradithiophene/pentacene analogy, indacene-dithiophenes have smaller energy level gaps than their purely hydrocarbon indenofluorene analogues, which is attributable to the increased paratropicity of the indacene core due to thiophene fusion. X-ray crystal packing reveals short intramolecular contact distances between LUMO-rich regions. Combined, these results suggest that 7a–d could make excellent candidates for electronic applications. Future work will consist of exploring derivatization and oligomerization/polymerization of the IDT structure as well as device construction to test their performance as organic semiconductors.

## Supplementary Material

SC-005-C3SC53181C-s001

SC-005-C3SC53181C-s002
