# Supplementary material for: Synthesis and properties of fully-conjugated indacenedithiophenes
Source: Chem Sci. 2013 Dec 10;5(3):1008–14. doi: 10.1039/c3sc53181c (PMC3979547; doi:10.1039/c3sc53181c)
Supplement: SC-005-C3SC53181C-s001 [file SC-005-C3SC53181C-s001.pdf]

Electronic Supplementary Material (ESI) for Chemical Science
This journal is © The Royal Society of Chemistry 2013

Electronic Supplementary Information for:

Synthesis and Properties of Fully Conjugated Indacenedithiophenes

Brian S. Young, Daniel T. Chase, Jonathan L. Marshall, Chris L. Vonnegut, Lev N. Zakharov and Michael M. Haley*

Department of Chemistry & Biochemistry and the Materials Science Institute
University of Oregon, Eugene, Oregon 97403-1253 US

haley@uoregon.edu

Table of Contents — Page

Experimental Details S2
Computational Details S10
References S19
Copies of ^{1}H and ^{13}C NMR Spectra S20

Electronic Supplementary Material (ESI) for Chemical Science
This journal is © The Royal Society of Chemistry 2013

Experimental Details

General Information. ^{1}H and ^{13}C NMR spectra were recorded in $CDCl_3$ using a Varian Inova 300 (^{1}H: 299.93 MHz, ^{13}C: 75.42 MHz), 500 (^{1}H: 500.11 MHz, ^{13}C: 125.75 MHz), or 600 (^{1}H: 599.90 MHz, ^{13}C: 150.88 MHz) spectrometer. Chemical shifts (δ) are expressed in ppm relative to the residual chloroform (^{1}H: 7.26 ppm, ^{13}C: 77.16 ppm) or DMSO (^{1}H: 2.50, ^{13}C: 39.52) reference. IR spectra were recorded on a Nicolet 6700 FTIR spectrometer with a Smart iTR ATR accessory. UV-Vis spectra were recorded on a HP 8453 UV-Vis spectrometer. High resolution mass spectra were recorded on a Waters LCT Premier ESI-MS in positive mode. Dry THF was distilled from a sodium and benzophenone still under N_2. All reagents were purchased from commercial suppliers and used as received unless otherwise indicated.

Synthesis

General Procedure A: Stille cross-coupling. Dibromide **10**[1] (1 equiv), $Pd(PPh_3)_4$ (3 mol%), and toluene (40 mL) were sparged with Ar for 45 min in a screwtop pressure reaction vessel. In a separate flask, the appropriate stannane (2.5 equiv) and toluene (10 mL) were also sparged with Ar for 45 min. After the stannane solution was transferred via cannula into the dibromide solution, the reaction vessel was sealed and stirred at 110 °C for 24 h. The reaction mixture was cooled, filtered through a short pad of silica eluting with DCM, and evaporated to dryness under reduced pressure to give the crude product which was purified by recrystallization (DCM:hexanes).

General Procedure B: Ester hydrolysis. The diester (1 equiv) and KOH (16 equiv) were refluxed in a 4:1 EtOH:H_2O mixture (0.023 M) for 24 h. The reaction was cooled, and the EtOH was evaporated under reduced pressure. The reaction mixture was then cooled in an ice bath and

the diacid was precipitated by careful addition of conc. HCl. The resulting solid was collected by vacuum filtration, washed with H_2O, oven dried, and used without further purification.

General Procedure C: Acid chloride formation/Friedel-Crafts acylation. To a suspension of the diacid (1 equiv) in dry DCM (0.065 M) was added oxalyl chloride (4 equiv), followed by dropwise addition of DMF (2 equiv). After stirring overnight, the reaction mixture was evaporated to dryness under reduced pressure yielding the crude diacid chloride, which was used without further purification. The crude acid chloride was dissolved in dry DCM (0.032 M), cooled to 0 °C, and $AlCl_3$ (4.7 equiv) was added. The reaction mixture was warmed to rt, stirred overnight, and then poured into an HCl-ice mixture. The solid dione was then collected by vacuum filtration, washed with HCl and H_2O, and dried under reduced pressure.

General Procedure D: Diol formation/reductive dearomatization. A stirred suspension of dione (1 equiv) in dry THF (30 mL) was cooled to –78 °C. In a separate flask, a stirred solution of MesBr (6 eq) in dry THF (10 mL) was cooled to –78 °C. BuLi (2.5 M in hexanes, 5 equiv) was added, and the reaction was stirred for 20 min after which it was transferred by cannula into the flask containing dione. This reaction mixture was warmed to rt and stirred overnight. The reaction was quenched with aq. NH_4Cl soln, extracted with DCM, dried ($MgSO_4$), and filtered through a short pad of silica to yield the crude diol which was used without further purification.

The crude diol was dissolved in toluene (40 mL) and sparged with Ar for 20 min. Anhydrous $SnCl_2$ (4 equiv) was added to the stirring solution, and the reaction was monitored by TLC. After completion, the reaction mixture was filtered through a short pad of silica, and the solvent removed under reduced pressure to give the crude IDT, which was purified by column chromatography on silica gel.

Preparation of IDT 7a

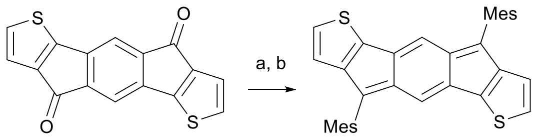

8a **7a**

Figure S1. Synthesis of IDT **7a**. *Reagents and conditions*: (a) MesBr, BuLi, THF, –78 °C; (b) $SnCl_2$, toluene, rt

Dione 8a. Dione **8a** was prepared as described in reference 2; spectroscopic data matched those previously reported. UV-Vis (DMSO) λ_{max} (ε): 293 (46,300), 566 (1,800) nm.

IDT 7a. Dione **8a** (0.075 g, 0.25 mmol) was reacted according to General Procedure D to give IDT **7a** (0.038 g, 30%) as a magenta solid. ^{1}H NMR (300 MHz, $CDCl_3$) δ 6.94 (s, 4H), 6.74 (d, J = 4.7 Hz, 2H), 6.24 (d, J = 4.7 Hz, 2H), 6.05 (s, 2H), 2.33 (s, 6H), 2.27 (s, 12H); ^{13}C NMR (150 MHz, $CDCl_3$) δ 150.9, 145.5, 142.6, 137.7, 136.8, 136.4, 133.0, 130.4, 128.4, 126.9, 122.9, 120.6, 21.3, 20.7; UV-Vis (DMSO) λ_{max} (ε): 340 (22,100), 561 (9,000) nm; HRMS (ESI+) for $C_{34}H_{28}S_2$ $(M+H)^+$: calcd 501.1711, found 501.1729.

Electronic Supplementary Material (ESI) for Chemical Science
This journal is © The Royal Society of Chemistry 2013

Preparation of IDBT 7b

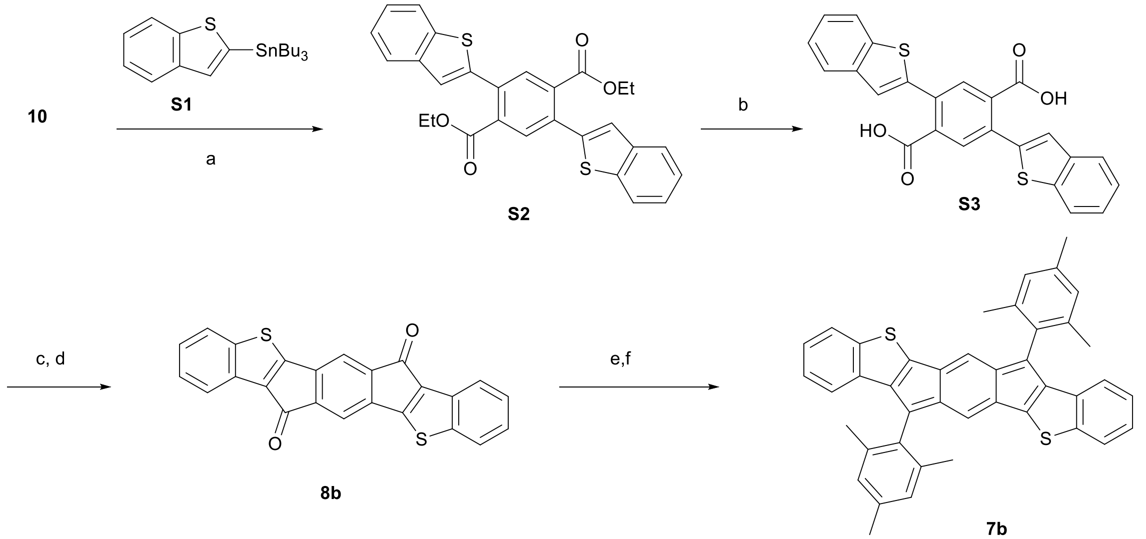

Figure S2. Synthesis of **7b**. *Reagents and conditions*: (a) $Pd(PPh_3)_4$, toluene, 110 °C; (b) KOH, EtOH,H_2O, reflux; (c) oxalyl chloride, DMF, DCM, rt; (d) $AlCl_3$, DCM, 0 °C; (e) MesBr, BuLi, THF, –78 °C; (f) $SnCl_2$, toluene, rt

Diester S2. Dibromide **10** (2.44 g, 6.4 mmol) and stannane **S1** were reacted according to General Procedure A to give diester **S2** (2.70 g, 86%) as a white solid. Spectroscopic data for this compound matched that which was previously reported in reference 1.

Diacid S3. Diester **S2** (2.70 g, 5.5 mmol) was reacted according to General Procedure B to give diacid **S3** (2.17 g, 91%) as a yellow solid. ^{1}H NMR (300 MHz, DMSO-d_6) δ 13.57 (br s, 2H), 8.07-8.00 (m, 2H), 7.94-7.87 (m, 2H), 7.90 (s, 2H), 7.59 (s, 2H), 7.46-7.39 (m, 4H); ^{13}C NMR (150 MHz, DMSO-d_6) δ 168.3, 140.1, 139.8, 139.8, 135.0, 132.5, 131.0, 124.9, 124.8, 124.1, 123.8, 122.3; HRMS (ESI+) for $C_{24}H_{14}O_4S_2$ $(M+H)^+$: calcd 431.0412, found 431.0404.

Dione 8b. Diacid **S3** was reacted according to General Procedure C to give dione **8b** (1.58 g, 80%) as a poorly soluble green solid. UV-Vis (DMSO) λ_{max} (ε): 307 (90,200), 341 (26,300), 601 (2,200) nm; HRMS (ESI+) for $C_{24}H_{10}O_2S_2$ $(M+H)^+$: calcd 395.0200, found 395.0216.

Electronic Supplementary Material (ESI) for Chemical Science
This journal is © The Royal Society of Chemistry 2013

IDT 7b. Dione **8b** (0.100 g, 0.25 mmol) was reacted according to General Procedure D to give IDT **7b** (0.035 g, 23%) as a blue solid. ^{1}H NMR (300 MHz, $CDCl_3$) δ 7.47 (d, *J* = 6.9 Hz, 2H), 7.04-6.93 (m, 4H), 6.99 (s, 4H), 6.59 (d, *J* = 6.9 Hz, 2H), 6.07 (s, 2H), 2.38 (s, 6H) 2.33 (s, 12H); ^{13}C NMR (150 MHz, $CDCl_3$) δ 148.1, 147.9, 144.0, 143.2, 137.9, 137.0, 136.8, 133.2, 131.8, 130.0, 128.4, 126.0, 125.6, 124.1, 123.8, 120.8, 21.4, 20.7; UV-Vis (DMSO) λ_{max} (ε): 373 (32,100), 624 (13,800) nm; HRMS (ESI+) for $C_{42}H_{32}S_2$ $(M+H)^+$: calcd 601.2024, found 601.2002.

Electronic Supplementary Material (ESI) for Chemical Science
This journal is © The Royal Society of Chemistry 2013

Preparation of IDT 7c

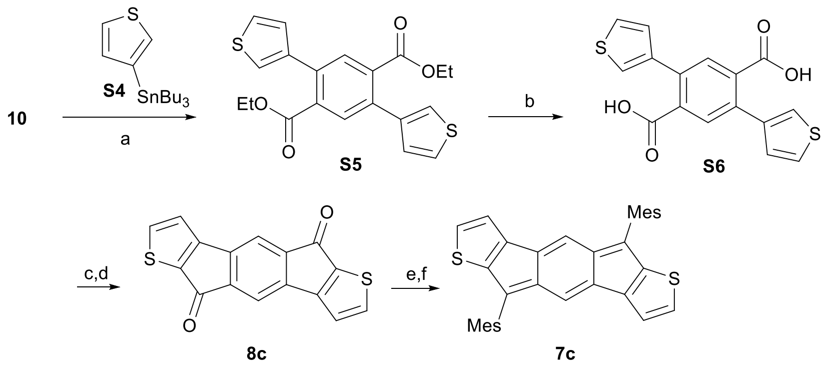

Figure S3. Synthesis of **7c**. *Reagents and conditions*: (a) $Pd(PPh_3)_4$, toluene, 110 °C; (b) KOH, EtOH, H_2O, reflux; (c) oxalyl chloride, DMF, DCM, rt; (d) $AlCl_3$, DCM, 0 °C; (e) MesBr, BuLi, THF, –78 °C; (f) $SnCl_2$, toluene, rt

Diester S5. Dibromide **10** (1.00 g, 2.6 mmol) and stannane **S4** were reacted according to General Procedure A to give diester **S5** (1.00 g, 98%) as a white solid. ^{1}H NMR (300 MHz, $CDCl_3$) δ 7.80 (s, 2H), 7.39-7.34 (m, 2H), 7.32-7.30 (m, 2H), 7.13 (dd, *J* = 4.8, 1.2 Hz, 2H), 4.19 (q, *J* = 7.2 Hz, 4H), 1.13 (t, *J* = 7.2 Hz, 6H); ^{13}C NMR (150 MHz, $CDCl_3$) δ 168.1, 140.3, 135.6, 133.7, 131.6, 128.5, 125.5, 123.0, 61.6, 14.0; HRMS (ESI+) for $C_{20}H_{18}O_4S_2$ $(M+H)^+$: calcd 387.0725, found 387.0730.

Diacid S6. Diester **S5** (0.43 g, 1.2 mmol) was reacted according to General Procedure B to give diacid **S6** (0.35 g, 95%) as a white solid. ^{1}H NMR (300 MHz, DMSO-d_6) δ 13.24 (br s, 2H), 7.70 (s, 2H), 7.65 (br s, 2H), 7.63-7.60 (m, 2H), 7.22 (d, *J* = 4.8 Hz, 2H); ^{13}C NMR (150 MHz, DMSO-d_6) δ 169.1, 139.5, 134.1, 133.6, 130.2, 128.2, 126.2, 123.5; HRMS (ESI+) for $C_{16}H_{10}O_4S_2$ $(M+H)^+$: calcd 331.0099, found 331.0089.

Dione 8c. Diacid **S6** (0.36 g, 1.1 mmol) was reacted according to General Procedure C to give dione **8c** (0.23 g, 70%) as a green solid. ^{1}H NMR (300 MHz, $CDCl_3$) δ 7.81 (d, *J* = 4.4 Hz, 2H), 7.34 (s, 2H), 7.13 (d, *J* = 4.4 Hz, 2H); ^{13}C NMR (150 MHz, $CDCl_3$) δ 184.4, 158.0, 142.4,

140.7, 140.3, 136.3, 120.5, 115.6; UV-Vis (DMSO) λ_{max} (ε): 279 (83,700), 312 (11,600), 370 (7,600) 551 (1,100) nm; HRMS (ESI+) for $C_{16}H_6O_2S_2$ $(M+H)^+$: calcd 294.9887, found 294.9896.

IDT 7c. Dione **8c** (0.100 g, 0.34 mmol) was reacted according General Procedure D to give IDT **7c** (0.036 g, 21%) as a purple solid. ^1H NMR (300 MHz, $CDCl_3$) δ 6.94 (s, 4H), 6.89 (d, *J* = 4.8 Hz, 2H), 6.57 (d, *J* = 4.8 Hz, 2H), 6.06 (s, 2H), 2.33 (s, 6H), 2.32 (s, 12H); ^{13}C NMR (150 MHz, $CDCl_3$) δ 146.8, 146.5, 145.0, 137.9, 136.9, 135.9, 133.2, 130.5, 129.9, 128.4, 123.3, 120.1, 21.3, 20.6; UV-Vis (DMSO) λ_{max} (ε): 307 (18,600), 592 (13,200) nm; HRMS (ESI+) for $C_{34}H_{28}S_2$ $(M+H)^+$: calcd 501.1711, found 501.1692.

Electronic Supplementary Material (ESI) for Chemical Science
This journal is © The Royal Society of Chemistry 2013

Preparation of IDT 7d

Diester 12. Dibromide **10** (1.00 g, 2.6 mmol) and stannane **11** (2.75 g, 6.5 mmol) were reacted according to General Procedure A to give diester **12** (0.825 g, 64%) as a white solid. ^{1}H NMR (300 MHz, $CDCl_3$) δ 8.06 (s, 2H), 7.94-7.90 (m, 2H), 7.56-7.51 (m, 2H), 7.44 (s, 2H), 7.39-7.35 (m, 4H), 3.91 (q, *J* = 7.2 Hz, 4H), 0.71 (t, *J* = 7.2 Hz, 6H); ^{13}C NMR (150 MHz, $CDCl_3$) δ 167.2, 139.8, 139.0, 136.2, 135.6, 134.8, 133.4, 124.6, 124.6, 124.1, 122.9, 122.5, 61.5, 13.4; HRMS (ESI+) for $C_{28}H_{22}O_4S_2$ $(M+H)^+$: calcd 487.1014, found 487.1024.

Diacid 13. Diester **12** (0.524 g, 1.1 mmol) was reacted according to General Procedure B to give diacid **13** (0.373 g, 80%) as a yellow solid. ^{1}H NMR (300 MHz, DMSO-d_6) δ 13.12 (br s, 2H), 8.09-8.06 (m, 2H), 7.91 (s, 2H), 7,84 (s, 2H), 7.55-7.51 (m, 2H), 7.45-7.39 (m, 4H); ^{13}C NMR (150 MHz, DMSO-d_6) δ 167.7, 139.2, 138.4, 135.4, 135.1, 134.4, 132.5, 125.1, 124.6, 124.4, 123.1, 122.0; HRMS (ESI+) for $C_{24}H_{14}O_4S_2$ $(M+H)^+$: calcd 431.0412, found 431.0420.

Dione 8d. Diacid **13** (0.37 g, 0.89 mmol) was reacted according to General Procedure C to give dione **8d** (0.299 g, 88%) as a poorly soluble blue solid. UV-Vis (DMSO) λ_{max} (ε): 292 (69,500), 385 (10,500), 609 (1,600) nm; HRMS (ESI+) for $C_{24}H_{10}O_2S_2$ $(M+H)^+$: calcd 395.0200, found 395.0197.

IDT 7d. Dione **8d** (0.100 g, 0.25 mmol) was reacted according to General Procedure D to give IDT **7d** (0.039 g, 26%) as a blue solid. ^{1}H NMR (300 MHz, $CDCl_3$) δ 7.45 (d, *J* = 7.5 Hz, 2H), 7.35 (d, *J* = 7.8 Hz, 2H), 7.13-7.06 (m, 4H), 6.97 (s, 4H), 6.02 (s, 2H), 2.41 (s, 12H), 2.36 (s, 6H); ^{13}C NMR (150 MHz, $CDCl_3$) δ 148.1, 147.0, 146.6, 141.1, 138.1, 137.6, 136.7, 135.0, 133.4, 129.4, 128.6, 125.4, 124.5, 124.4, 124.1, 122.0, 21.3, 20.8; UV-Vis (DMSO) λ_{max} (ε): 322 (19,700), 632 (14,800) nm; HRMS (ESI+) for $C_{42}H_{32}S_2$ $(M+H)^+$: calcd 601.2024, found 601.2041.

Calculations

DFT calculations were performed using the Gaussian09 suite of programs.[3] Harmonic frequency analysis at the same level of theory as the minimization were used to confirm minimized structures.

Cartesian Coordinates

7a'
B3LYP/6-311+G** = -1411.03315213 au
B3LYP/6-311+G** Zero Point Corrected Energy = -1410.849816 au
NIMAG = 0

C	-1.37152	0.33592	-0.00002
C	-0.91845	-1.06162	0.00005
C	0.47525	-1.36727	0.00004
C	1.37152	-0.33592	0.00001
C	0.91845	1.06162	-0.00006
C	-0.47525	1.36727	-0.00007
C	2.81856	-0.26196	-0.00005
C	3.21483	1.07864	-0.00006
C	2.02524	1.89588	-0.00007
C	-2.81856	0.26196	0.00002
C	-3.21483	-1.07864	0.00004
C	-2.02524	-1.89588	0.00008
S	-4.17334	1.32595	-0.00004
C	-5.27965	-0.03668	0.00005
C	-4.63189	-1.23930	0.00000
S	4.17334	-1.32595	-0.00006
C	5.27965	0.03668	0.00009
C	4.63189	1.23930	0.00007
H	0.80022	-2.40331	0.00005
H	-0.80022	2.40331	-0.00009
H	-6.34177	0.15430	0.00008
H	-5.15099	-2.18876	0.00001
H	6.34177	-0.15431	0.00017
H	5.15099	2.18876	0.00016
H	2.00146	2.97837	0.00040
H	-2.00145	-2.97837	0.00005

7b'
B3LYP/6-311+G** = -1718.40774297 au
B3LYP/6-311+G** Zero Point Corrected Energy = -1718.131102 au
NIMAG = 0

C	1.31245	0.50256	0.00007
C	1.04747	-0.93419	-0.00008
C	-0.28135	-1.41637	-0.00025
C	-1.31245	-0.50256	-0.00028
C	-1.04747	0.93419	-0.00014
C	0.28135	1.41637	0.00006
C	2.74552	0.62135	0.00009
C	3.32333	-0.65831	0.00006
C	-2.74552	-0.62135	-0.00023
C	-3.32333	0.65831	-0.00016
C	2.27142	-1.61782	0.00011
S	3.91599	1.89811	0.00008
C	5.24039	0.70958	0.00004
C	4.76304	-0.62587	0.00004
C	-2.27142	1.61782	-0.00042
S	-3.91599	-1.89811	-0.00009
C	-5.24039	-0.70958	0.00007
C	-4.76304	0.62587	0.00002
C	6.60016	1.00649	0.00003
C	7.50893	-0.04828	0.00002
C	-6.60016	-1.00649	0.00022
C	-7.50893	0.04828	0.00031
C	7.05941	-1.37577	0.00002
C	5.70142	-1.66847	0.00003
C	-7.05941	1.37577	0.00024
C	-5.70142	1.66847	0.00009
H	-0.47245	-2.48539	-0.00025
H	0.47245	2.48539	0.00006
H	6.94662	2.03351	0.00004
H	8.57205	0.16199	0.00001
H	-6.94662	-2.03351	0.00027
H	-8.57205	-0.16199	0.00043
H	7.78161	-2.18416	0.00001
H	5.36444	-2.69912	0.00003
H	-7.78161	2.18416	0.00030
H	-5.36444	2.69912	0.00003
H	2.38982	-2.69353	0.00017
H	-2.38982	2.69353	-0.00059

7c'
B3LYP/6-311+G** = -1411.03569727 au
B3LYP/6-311+G** Zero Point Corrected Energy = -1410.852206 au
NIMAG = 0

C	1.41416	0.10017	-0.00025
C	0.72871	-1.19000	-0.00016
C	-0.69487	-1.26512	-0.00003
C	-1.41416	-0.10017	-0.00003
C	-0.72871	1.19000	-0.00012
C	0.69487	1.26512	-0.00022
C	-2.84259	0.19780	0.00002
C	-2.96732	1.58562	0.00001
C	-1.68165	2.20624	-0.00004
C	2.84259	-0.19780	-0.00017
C	2.96732	-1.58563	-0.00013
C	1.68165	-2.20625	-0.00037
C	4.10234	0.47594	-0.00006
S	4.62477	-2.13477	-0.00003
C	5.17995	-0.45768	0.00005
C	-4.10235	-0.47594	0.00008
S	-4.62477	2.13477	0.00003
C	-5.17995	0.45768	0.00011
C	4.41100	1.85032	-0.00001
C	5.73346	2.26156	0.00012
C	6.77975	1.32342	0.00021
C	6.51003	-0.03963	0.00017
C	-4.41101	-1.85032	0.00013
C	-5.73346	-2.26155	0.00020
C	-6.77976	-1.32342	0.00023
C	-6.51003	0.03964	0.00018
H	-1.17878	-2.23620	-0.00003
H	1.17878	2.23619	-0.00022
H	3.61372	2.58382	-0.00008
H	5.96655	3.32015	0.00015
H	7.80839	1.66477	0.00031
H	7.31817	-0.76183	0.00024
H	-3.61373	-2.58382	0.00011
H	-5.96656	-3.32015	0.00023
H	-7.80839	-1.66476	0.00028
H	-7.31817	0.76184	0.00020
H	-1.48220	3.26911	-0.00011
H	1.48221	-3.26911	-0.00059

7d'

B3LYP/6-311+G** = -1718.40587489 au
B3LYP/6-311+G** Zero Point Corrected Energy = -1718.128887 au
NIMAG = 0

C	1.32591	-0.48869	-0.00016
C	1.03250	0.94436	-0.00005
C	-0.31597	1.40998	0.00010
C	-1.32591	0.48869	0.00014
C	-1.03250	-0.94436	0.00003
C	0.31597	-1.40998	-0.00014
C	-2.78092	0.60632	0.00015
C	-3.29325	-0.69644	0.00001
C	-2.23143	-1.65277	0.00022
C	2.78092	-0.60632	-0.00012
C	3.29325	0.69644	-0.00000
C	2.23143	1.65277	-0.00023
C	3.79432	-1.59482	-0.00014
S	5.02788	0.70415	0.00026
C	5.05144	-1.03966	-0.00014
C	-3.79432	1.59482	0.00018
S	-5.02788	-0.70415	-0.00027
C	-5.05144	1.03966	0.00015
H	-0.51960	2.47654	0.00011
H	0.51960	-2.47654	-0.00014
H	3.62849	-2.66412	-0.00022
H	6.00185	-1.55158	-0.00021
H	-3.62849	2.66412	0.00028
H	-6.00185	1.55158	0.00021
H	-2.33606	-2.72911	0.00021
H	2.33606	2.72911	-0.00014

8a

B3LYP/6-311+G** = -1560.35609332 au
B3LYP/6-311+G** Zero Point Corrected Energy = -1560.186107 au
NIMAG = 0

C	-1.33257	0.38693	-0.00005
C	-0.96315	-0.97470	-0.00009
C	0.36124	-1.38854	-0.00018
C	1.33262	-0.38687	-0.00016
C	0.96320	0.97472	-0.00007
C	-0.36125	1.38856	-0.00001
C	2.80113	-0.42114	-0.00040
C	3.33766	0.85003	-0.00041

C	2.21743	1.82902	-0.00015
C	-2.80117	0.42116	0.00020
C	-3.33763	-0.85002	0.00013
C	-2.21744	-1.82902	-0.00001
S	-4.02552	1.62610	0.00004
C	-5.27275	0.39566	0.00010
C	-4.75684	-0.87120	0.00000
S	4.02551	-1.62604	0.00016
C	5.27269	-0.39583	0.00037
C	4.75686	0.87110	0.00003
O	-2.26279	-3.03905	-0.00007
O	2.26277	3.03907	0.00010
H	0.60706	-2.44455	-0.00014
H	-0.60702	2.44459	0.00002
H	-6.30786	0.70135	0.00007
H	-5.36219	-1.76718	0.00004
H	6.30777	-0.70162	0.00054
H	5.36240	1.76694	0.00015

8b

B3LYP/6-311+G** = -1867.72868549 au
B3LYP/6-311+G** Zero Point Corrected Energy = -1867.464858 au
NIMAG = 0

C	1.29669	-0.48611	-0.00011
C	1.03489	0.89900	-0.00017
C	-0.25341	1.41326	-0.00008
C	-1.29670	0.48611	0.00003
C	-1.03490	-0.89899	0.00011
C	0.25341	-1.41326	0.00006
C	2.75868	-0.63326	-0.00000
C	3.39027	0.58613	0.00009
C	-2.75868	0.63326	0.00016
C	-3.39027	-0.58613	0.00033
C	2.35601	1.64813	-0.00009
S	3.85015	-1.97301	-0.00026
C	5.23312	-0.86033	-0.00018
O	2.49840	2.85228	-0.00001
C	4.81965	0.49799	0.00001
C	-2.35600	-1.64813	0.00019
S	-3.85016	1.97301	-0.00009
C	-5.23312	0.86033	-0.00004
C	-4.81964	-0.49799	0.00020
O	-2.49839	-2.85227	-0.00003
C	6.57976	-1.21920	-0.00009

C	7.52851	-0.20305	0.00012
C	-6.57976	1.21920	-0.00024
C	-7.52851	0.20304	-0.00018
C	7.13894	1.14612	0.00027
C	5.79925	1.50448	0.00022
C	-7.13894	-1.14613	0.00006
C	-5.79924	-1.50449	0.00026
H	-0.41939	2.48471	-0.00012
H	0.41939	-2.48470	0.00011
H	6.88256	-2.25965	-0.00024
H	8.58177	-0.45822	0.00015
H	-6.88257	2.25965	-0.00037
H	-8.58177	0.45821	-0.00030
H	7.89917	1.91865	0.00046
H	5.49718	2.54461	0.00036
H	-7.89916	-1.91866	0.00010
H	-5.49717	-2.54461	0.00045

8c
B3LYP/6-311+G** = -1560.35683245 au
B3LYP/6-311+G** Zero Point Corrected Energy = -1560.186550 au
NIMAG = 0

C	1.28519	0.52855	-0.00018
C	1.06140	-0.86141	-0.00060
C	-0.21164	-1.41796	-0.00064
C	-1.28518	-0.52845	-0.00048
C	-1.06139	0.86152	-0.00029
C	0.21165	1.41806	-0.00030
C	-2.74953	-0.74504	0.00009
C	-3.38322	0.48207	-0.00017
C	-2.39640	1.58396	-0.00051
C	2.74957	0.74507	-0.00053
C	3.38320	-0.48207	-0.00080
C	2.39634	-1.58393	-0.00042
C	3.65213	1.83520	0.00087
S	5.09857	-0.33059	0.00021
C	4.95692	1.39832	0.00121
C	-3.65202	-1.83524	0.00051
S	-5.09858	0.33049	0.00044
O	2.57239	-2.78217	-0.00005
O	-2.57253	2.78219	-0.00072
C	-4.95684	-1.39842	0.00084
H	-0.34125	-2.49432	-0.00022
H	0.34126	2.49443	-0.00084

H	3.37798	2.88147	0.00158
H	5.85342	2.00071	0.00186
H	-3.37780	-2.88148	0.00046
H	-5.85329	-2.00088	0.00124

8d
B3LYP/6-311+G** = -1867.72374476 au
B3LYP/6-311+G** Zero Point Corrected Energy = -1867.459933 au
NIMAG = 0

C	-1.39166	0.08896	-0.00002
C	-0.72884	-1.15294	0.00008
C	0.65458	-1.27387	0.00043
C	1.39167	-0.08892	0.00022
C	0.72885	1.15298	0.00000
C	-0.65456	1.27389	0.00010
C	2.84819	0.18233	0.00005
C	3.05176	1.53973	-0.00005
C	1.75923	2.26406	-0.00002
C	-2.84818	-0.18231	-0.00041
C	-3.05174	-1.53970	-0.00053
C	-1.75921	-2.26403	-0.00002
C	-4.06691	0.56633	-0.00014
S	-4.72035	-2.00928	-0.00013
C	-5.18960	-0.31073	-0.00005
C	4.06691	-0.56632	0.00002
S	4.72039	2.00927	0.00003
O	-1.54483	-3.45575	0.00034
O	1.54485	3.45578	-0.00001
C	5.18961	0.31072	-0.00009
C	-4.29407	1.95640	-0.00007
C	-5.59151	2.43680	0.00013
C	-6.68702	1.55439	0.00022
C	-6.49718	0.18021	0.00007
C	4.29401	-1.95640	0.00005
C	5.59144	-2.43684	-0.00001
C	6.68696	-1.55445	-0.00010
C	6.49718	-0.18026	-0.00018
H	1.11289	-2.25528	0.00047
H	-1.11290	2.25530	-0.00011
H	-3.45835	2.64518	-0.00020
H	-5.76850	3.50587	0.00026
H	-7.69493	1.95283	0.00042
H	-7.34431	-0.49548	0.00019
H	3.45826	-2.64514	0.00009

H	5.76842	-3.50590	0.00008
H	7.69487	-1.95292	-0.00008
H	7.34434	0.49538	-0.00019

4'
B3LYP/6-311+G** = -769.528277131 au
B3LYP/6-311+G** Zero Point Corrected Energy = -769.277761 au
NIMAG = 0

C	-1.35537	0.40169	-0.00005
C	-0.96718	-1.01430	0.00018
C	0.41896	-1.38731	0.00018
C	1.35536	-0.40169	-0.00034
C	0.96718	1.01430	-0.00031
C	-0.41896	1.38731	-0.00036
C	2.82209	-0.43277	0.00002
C	3.27036	0.91869	0.00040
C	2.10448	1.78624	0.00015
C	-2.82209	0.43278	-0.00007
C	-3.27035	-0.91869	0.00014
C	-2.10447	-1.78623	0.00030
C	-3.73894	1.47439	-0.00019
C	-5.10661	1.17263	-0.00014
C	-5.54790	-0.15306	0.00004
C	-4.63367	-1.20981	0.00020
C	3.73894	-1.47439	-0.00050
C	5.10661	-1.17264	-0.00035
C	5.54790	0.15305	0.00017
C	4.63368	1.20980	0.00052
H	0.69380	-2.43754	0.00054
H	-0.69381	2.43755	-0.00071
H	2.13801	2.86838	0.00040
H	-2.13800	-2.86837	0.00045
H	-3.41170	2.50869	-0.00028
H	-5.83209	1.97823	-0.00026
H	-6.61152	-0.36290	0.00008
H	-4.98219	-2.23706	0.00036
H	3.41169	-2.50869	-0.00095
H	5.83208	-1.97824	-0.00073
H	6.61152	0.36289	0.00027
H	4.98220	2.23705	0.00084

9

B3LYP/6-311+G** = -462.140152591 au
B3LYP/6-311+G** Zero Point Corrected Energy = -461.984624 au
NIMAG = 0

C	1.20912	-0.72064	-0.00001
C	1.19136	0.72871	-0.00001
C	-0.03447	1.44142	-0.00000
C	-1.20912	0.72063	-0.00001
C	-1.19136	-0.72870	-0.00001
C	0.03447	-1.44142	0.00000
C	-2.59642	1.12441	-0.00002
C	-3.37947	-0.01356	0.00003
C	-2.52144	-1.15948	-0.00001
C	2.59642	-1.12441	-0.00002
C	3.37947	0.01356	0.00003
C	2.52144	1.15948	-0.00000
H	-0.03871	2.52792	-0.00000
H	0.03870	-2.52792	0.00000
H	-2.95062	2.14657	-0.00002
H	-4.45961	-0.04467	0.00006
H	-2.85431	-2.18967	-0.00000
H	2.95062	-2.14657	-0.00002
H	4.45960	0.04466	0.00006
H	2.85431	2.18967	0.00000

Electronic Supplementary Material (ESI) for Chemical Science
This journal is © The Royal Society of Chemistry 2013

References

1. Wong, K.-T.; Chao, T.-C.; Chi, L.-C.; Chu, Y.-Y.; Balaiah, A.; Chiu, S.-F.; Liu, Y.-H.; Wang, Y. *Org. Lett.* **2006**, *8*, 5033–5036.

2. Zhang, W.; Smith, J.; Watkins, S. E.; Gysel, R.; McGehee, M.; Salleo, A.; Kirkpatrick, J.; Ashraf, S.; Anthopoulos, T.; Heeney, M.; McCulloch, I. *J. Am. Chem. Soc.* **2010**, *132*, 11437–11439.

3. Frisch, M. J.; Trucks, G. W.; Schlegel, H. B.; Scuseria, G. E.; Robb, M. A.; Cheeseman, J. R.; Scalmani, G.; Barone, V.; Mennucci, B.; Petersson, G. A.; Nakatsuji, H.; Caricato, M.; Li, X.; Hratchian, H. P.; Izmaylov, A. F.; Bloino, J.; Zheng, G.; Sonnenberg, J. L.; Hada, M.; Ehara, M.; Toyota, K.; Fukuda, R.; Hasegawa, J.; Ishida, M.; Nakajima, T.; Honda, Y.; Kitao, O.; Nakai, H.; Vreven, T.; Montgomery, Jr., J. A.; Peralta, J. E.; Ogliaro, F.; Bearpark, M.; Heyd, J. J.; Brothers, E.; Kudin, K. N.; Staroverov, V. N.; Kobayashi, R.; Normand, J.; Raghavachari, K.; Rendell, A.; Burant, J. C.; Iyengar, S. S.; Tomasi, J.; Cossi, M.; Rega, N.; Millam, J. M.; Klene, M.; Knox, J. E.; Cross, J. B.; Bakken, V.; Adamo, C.; Jaramillo, J.; Gomperts, R.; Stratmann, R. E.; Yazyev, O.; Austin, A. J.; Cammi, R.; Pomelli, C.; Ochterski, J. W.; Martin, R. L.; Morokuma, K.; Zakrzewski, V. G.; Voth, G. A.; Salvador, P.; Dannenberg, J. J.; Dapprich, S.; Daniels, A. D.; Farkas, Ö.; Foresman, J. B.; Ortiz, J. V.; Cioslowski, J.; Fox, D. J., *Gaussian 09, Revision A.02*, Gaussian, Inc., Wallingford, CT, **2009**.

Spectral Data

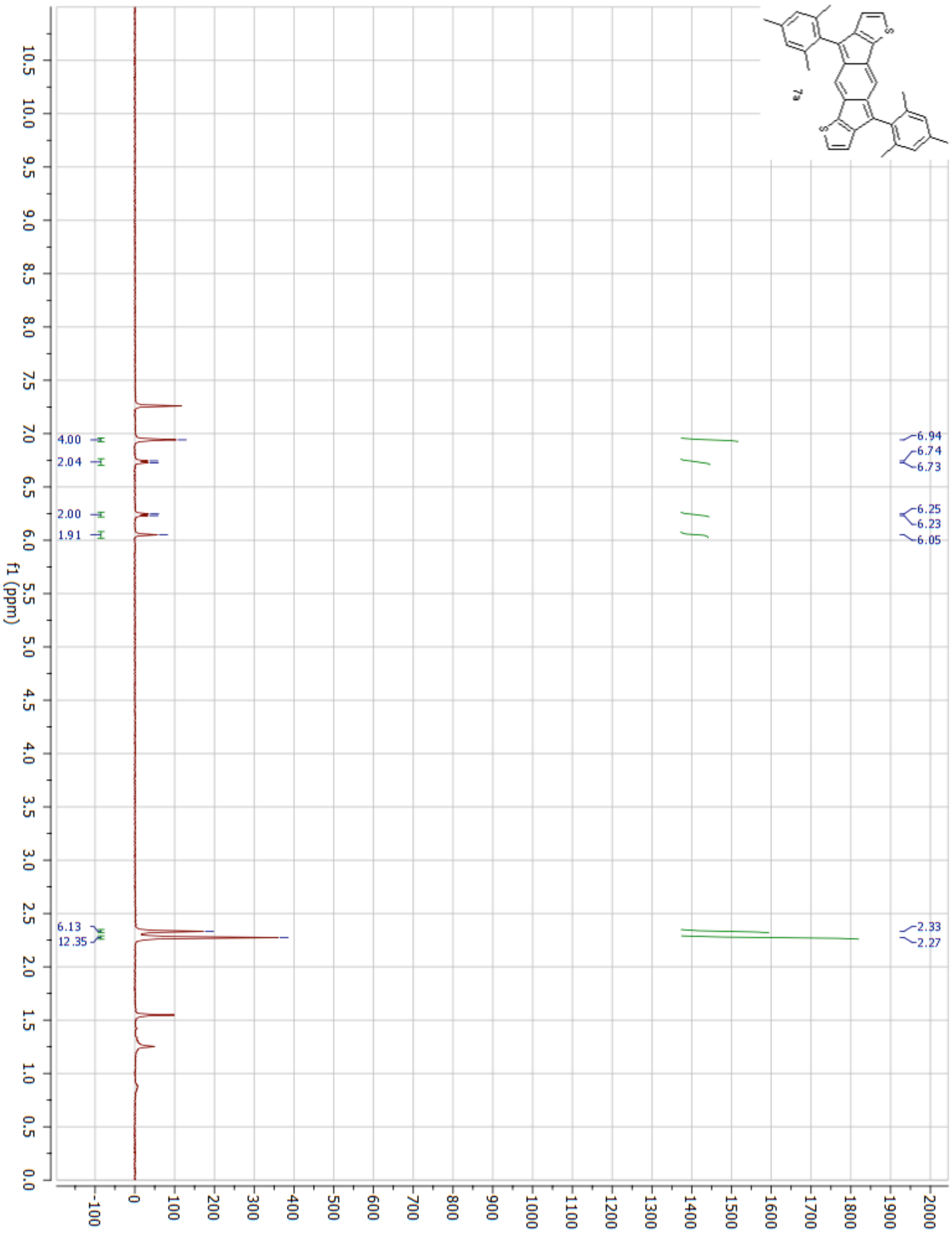

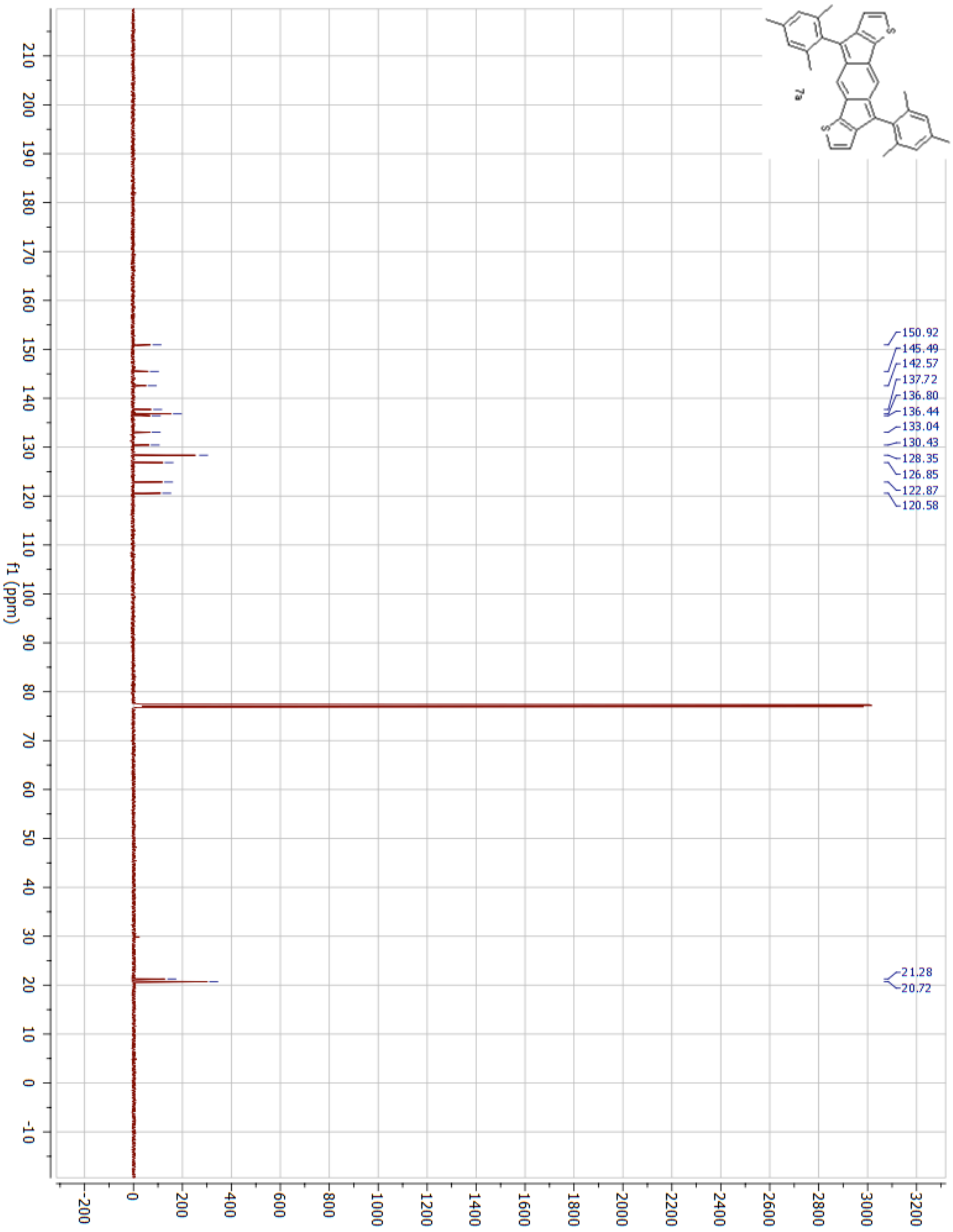
7a
150.92
145.49
142.57
137.72
136.80
136.44
133.04
130.43
128.35
126.85
122.87
120.58
21.28
20.72
f1 (ppm)

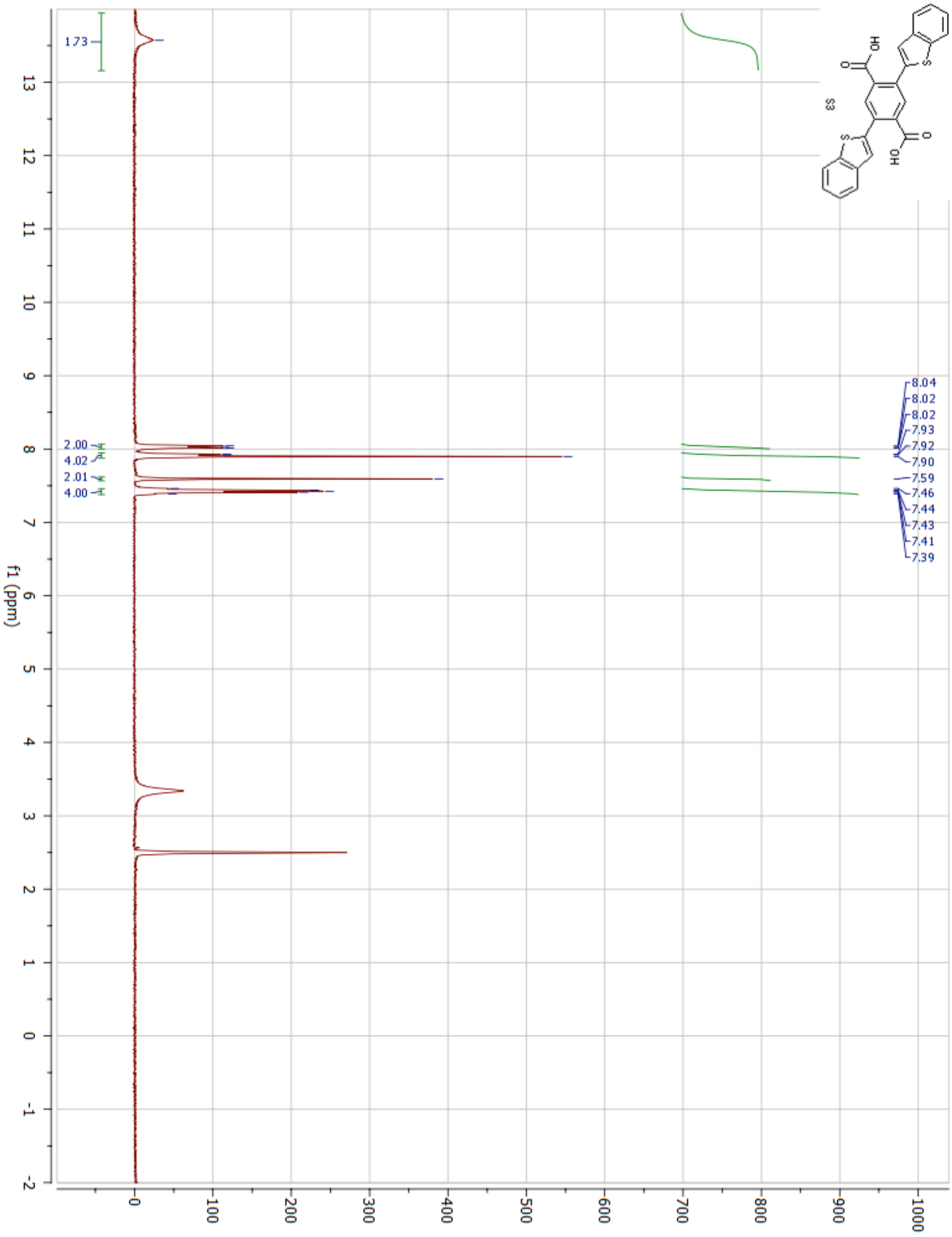
S3
8.04
8.02
8.02
7.93
7.92
7.90
7.59
7.46
7.44
7.43
7.41
7.39
1.73
2.00
4.02
2.01
4.00
f1 (ppm)

Electronic Supplementary Material (ESI) for Chemical Science
This journal is © The Royal Society of Chemistry 2013

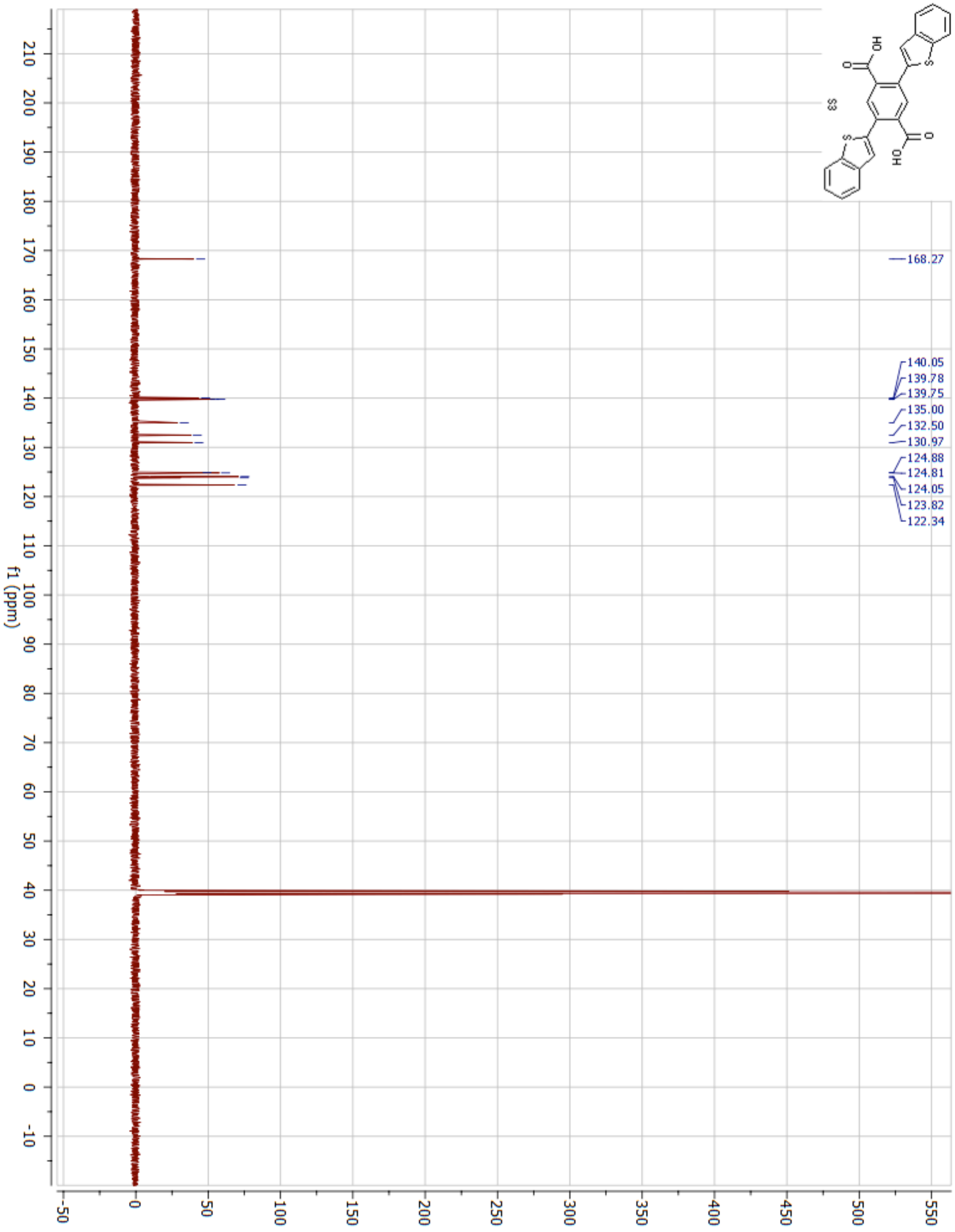

Electronic Supplementary Material (ESI) for Chemical Science
This journal is © The Royal Society of Chemistry 2013

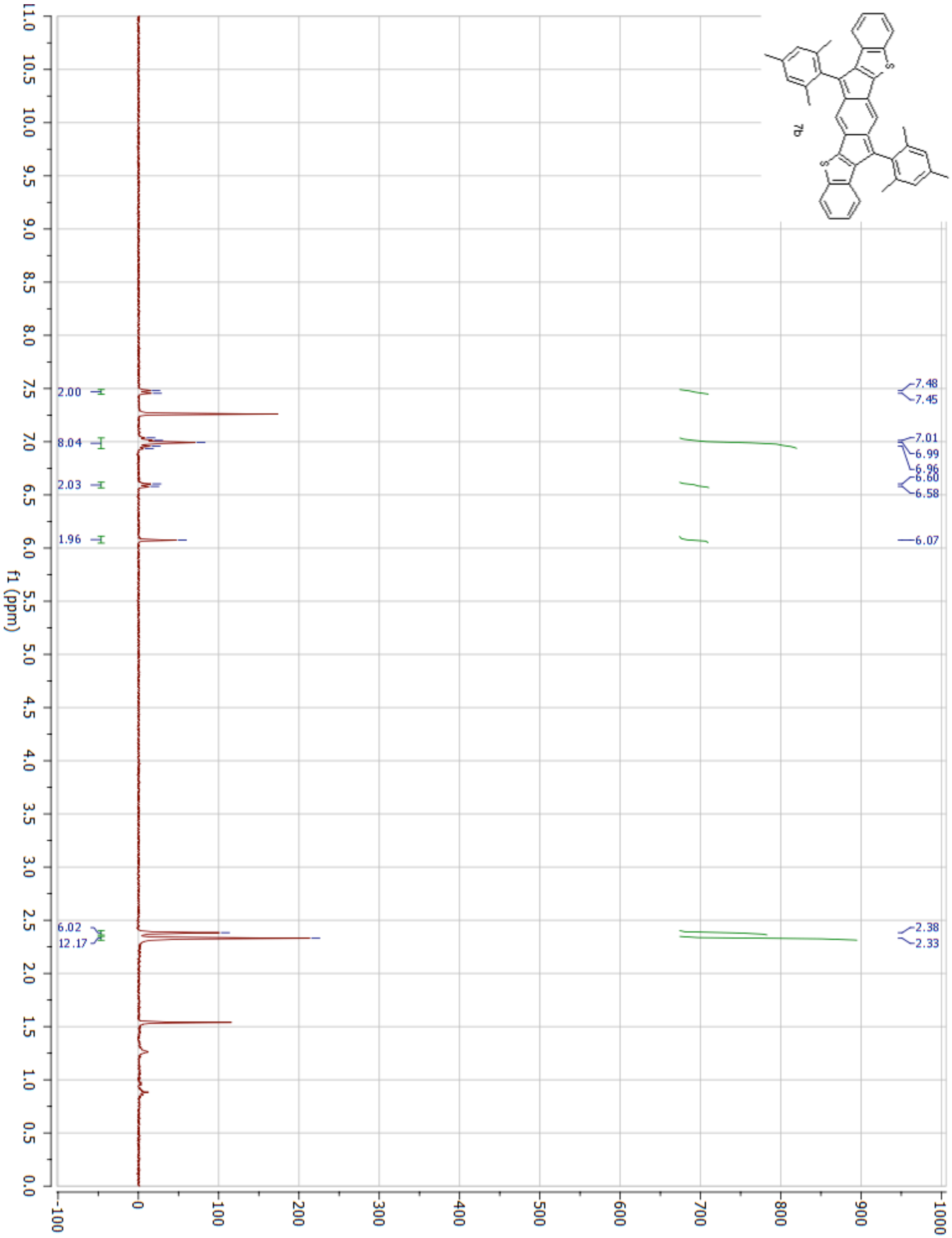

This journal is © The Royal Society of Chemistry 2013

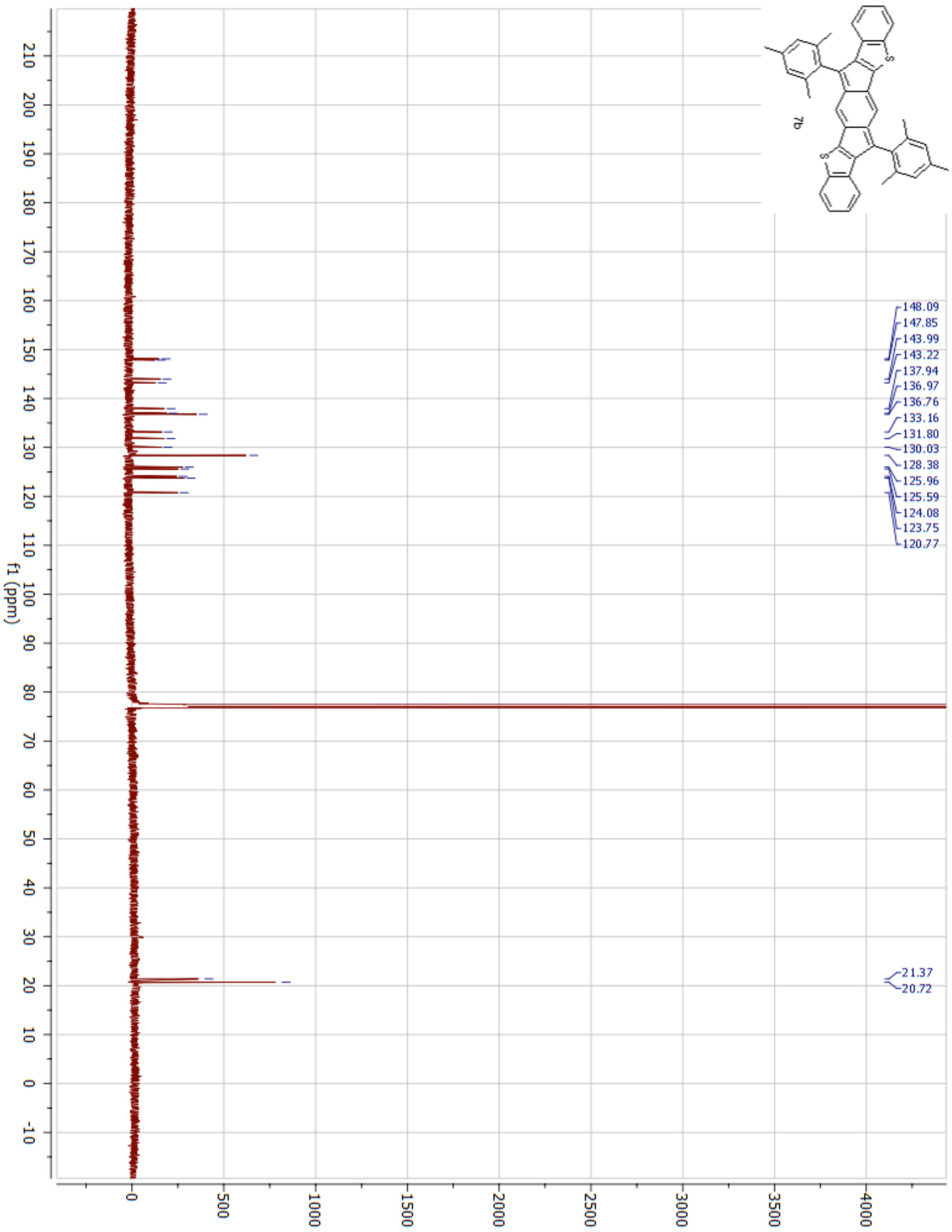

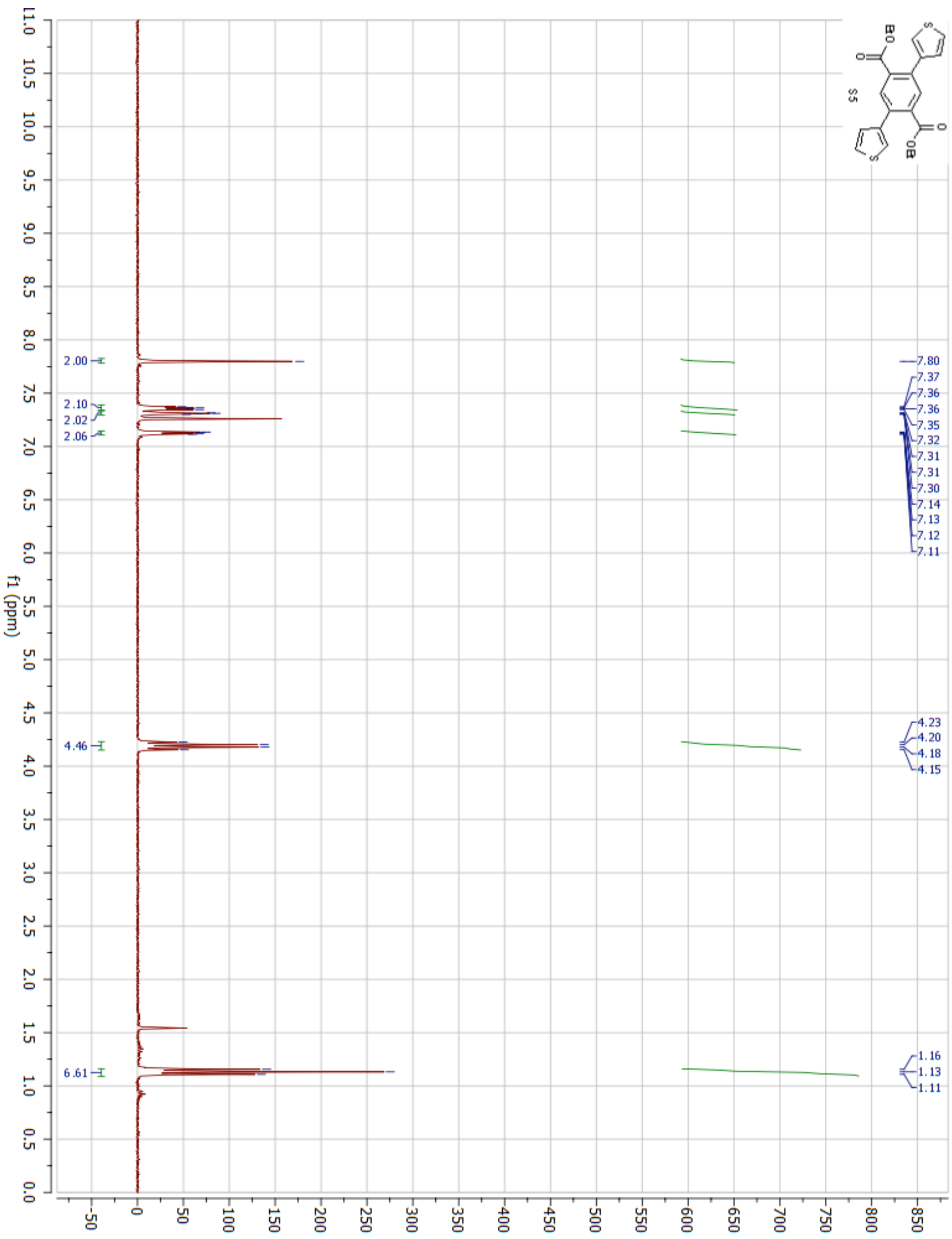
S5
OEt
O
S
f1 (ppm)
7.80
7.37
7.36
7.36
7.35
7.32
7.31
7.31
7.30
7.14
7.13
7.12
7.11
4.23
4.20
4.18
4.15
1.16
1.13
1.11
2.00
2.10
2.02
2.06
4.46
6.61

Electronic Supplementary Material (ESI) for Chemical Science
This journal is © The Royal Society of Chemistry 2013

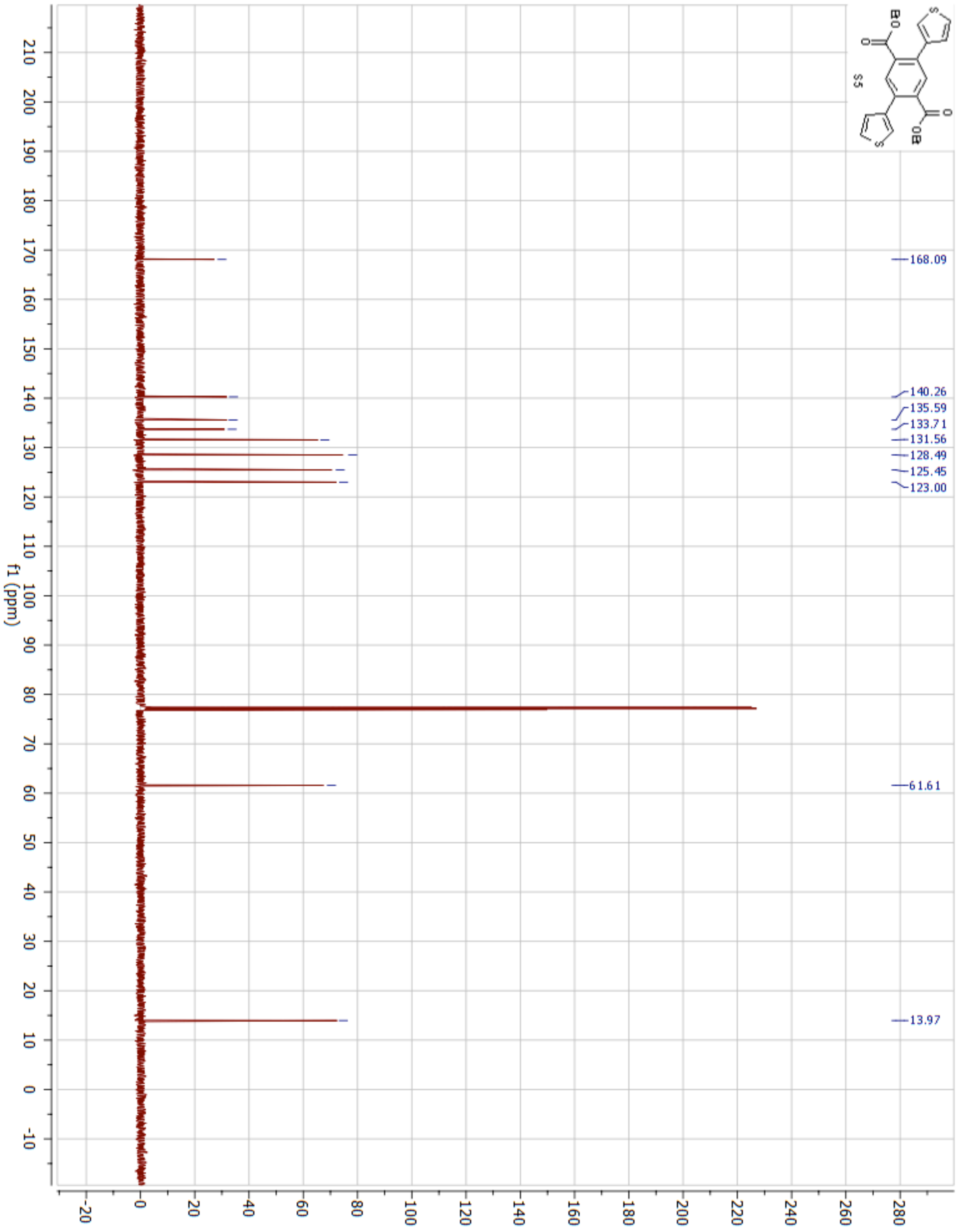

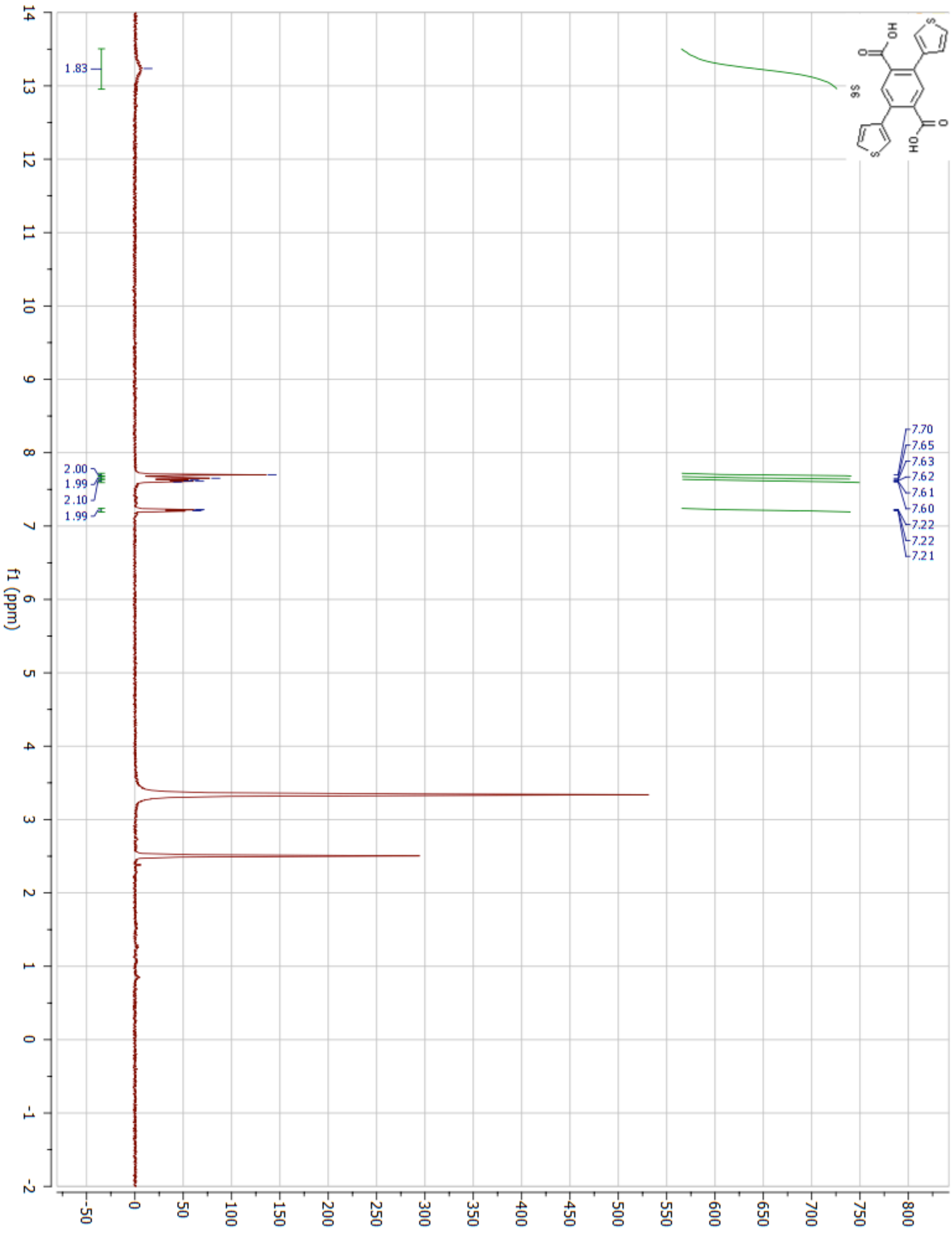
S6
7.70
7.65
7.63
7.62
7.61
7.60
7.22
7.22
7.21
1.83
2.00
1.99
2.10
1.99
f1 (ppm)

Electronic Supplementary Material (ESI) for Chemical Science
This journal is © The Royal Society of Chemistry 2013

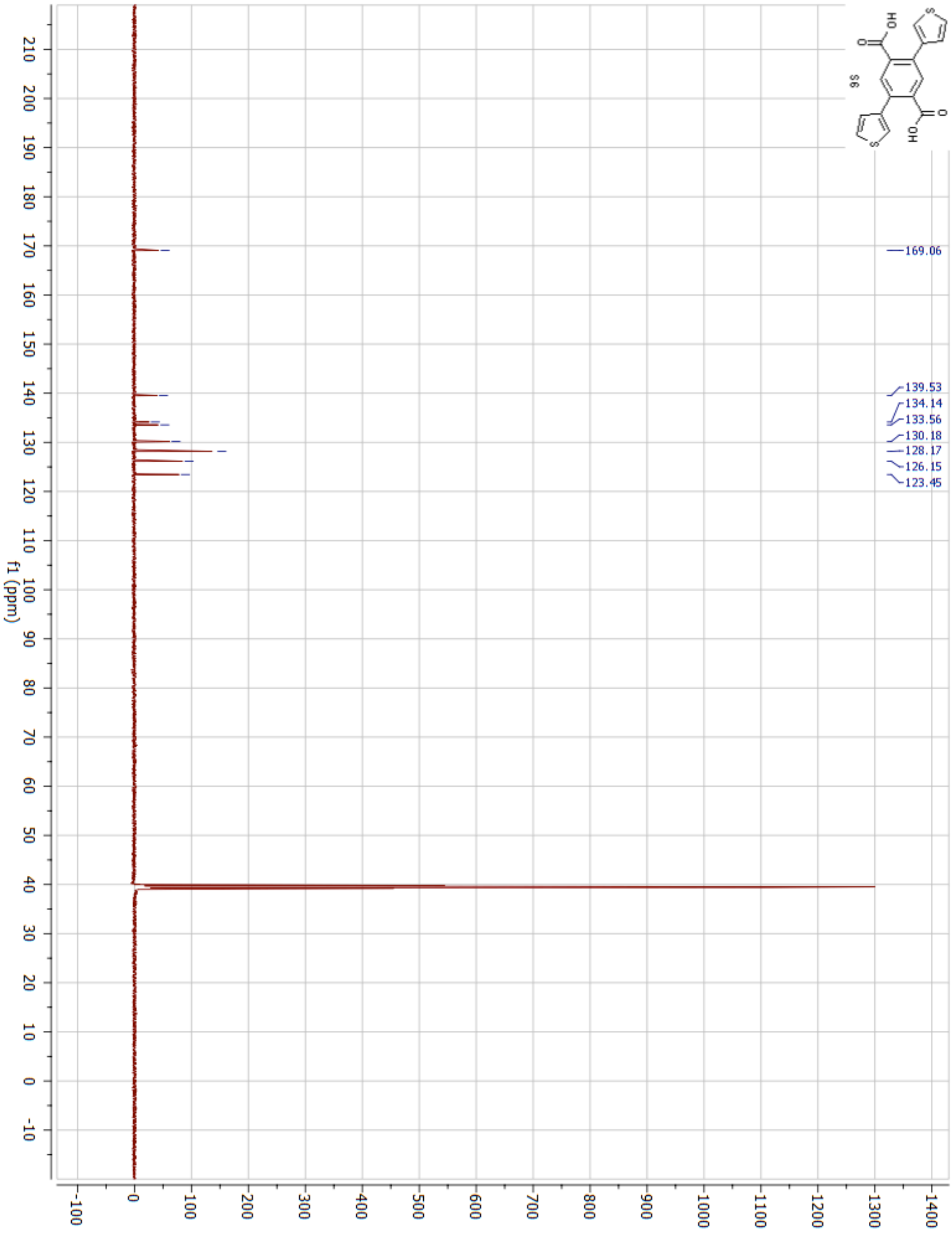

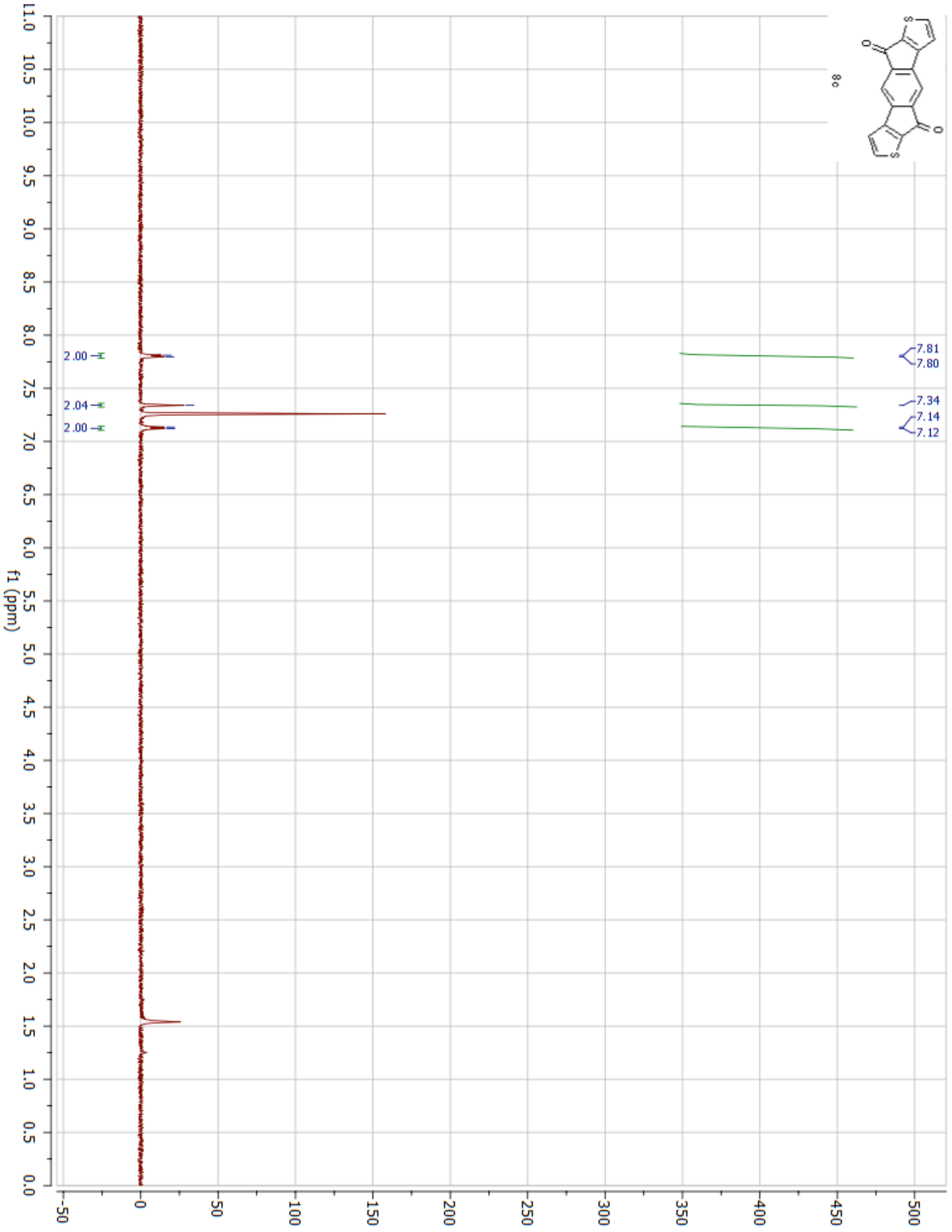
8c
f1 (ppm)
2.00
2.04
2.00
7.81
7.80
7.34
7.14
7.12

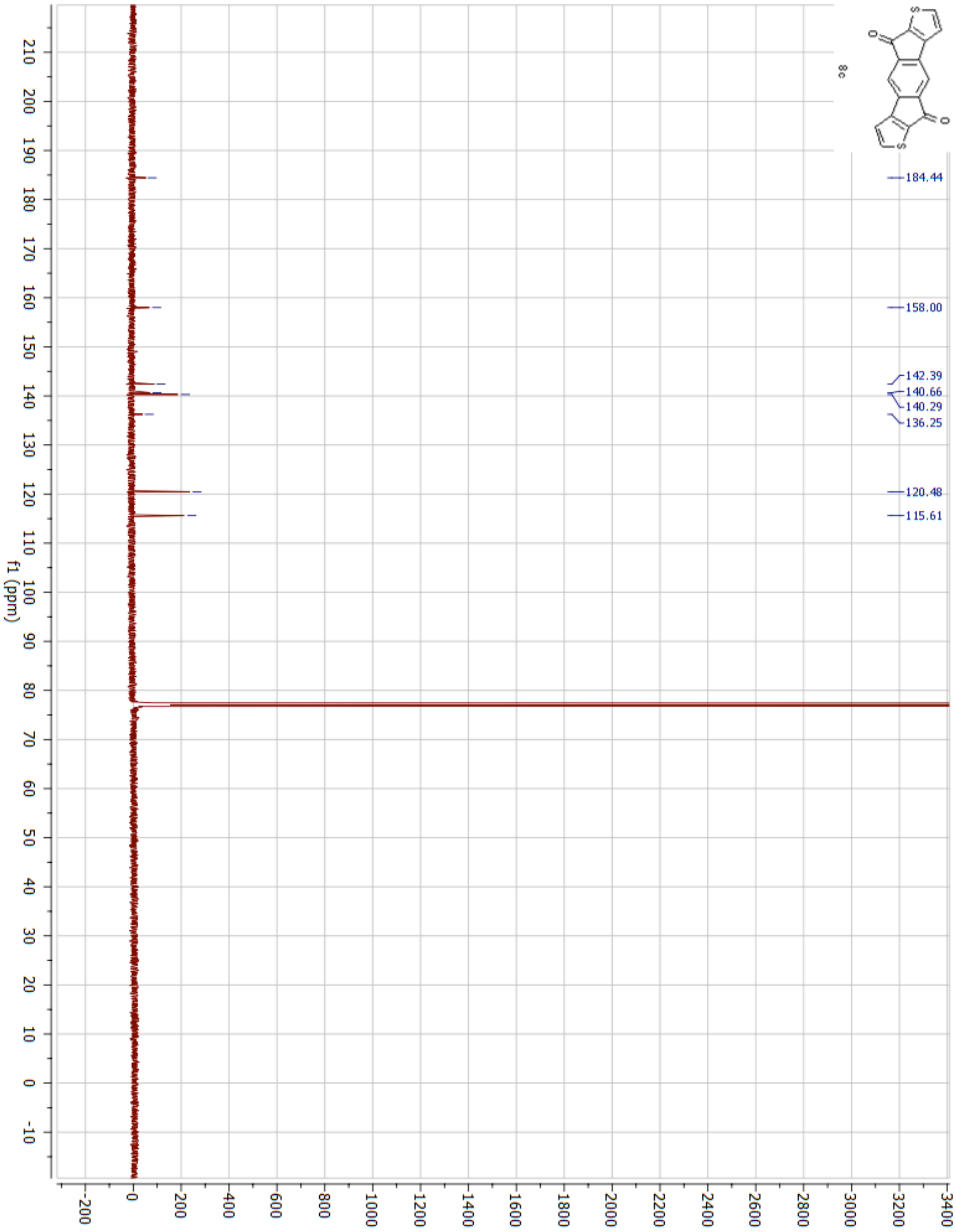
8c
184.44
158.00
142.39
140.66
140.29
136.25
120.48
115.61
f1 (ppm)

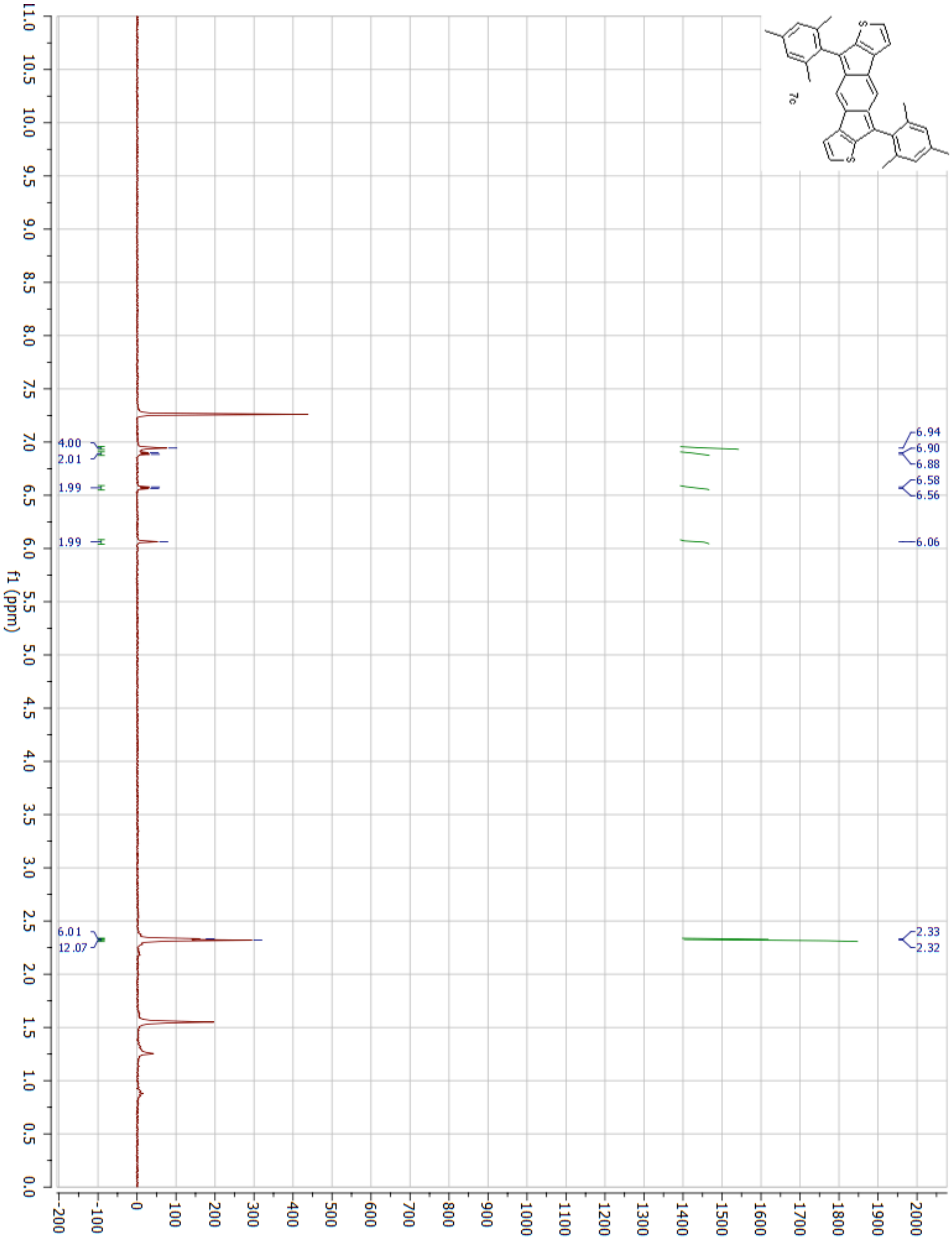
7c
f1 (ppm)
4.00
2.01
1.99
1.99
6.01
12.07
6.94
6.90
6.88
6.58
6.56
6.06
2.33
2.32

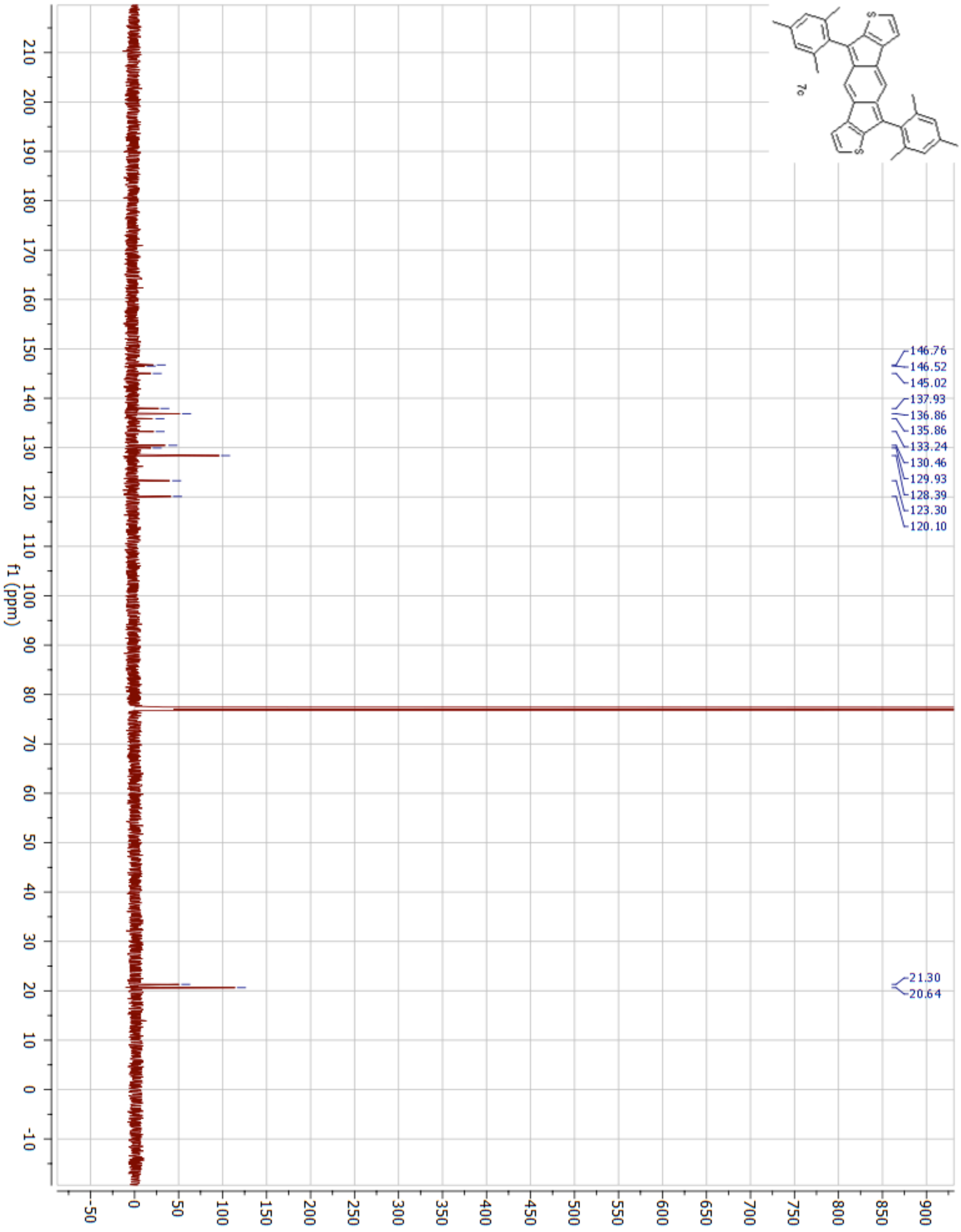
7c
146.76
146.52
145.02
137.93
136.86
135.86
133.24
130.46
129.93
128.39
123.30
120.10
21.30
20.64
f1 (ppm)

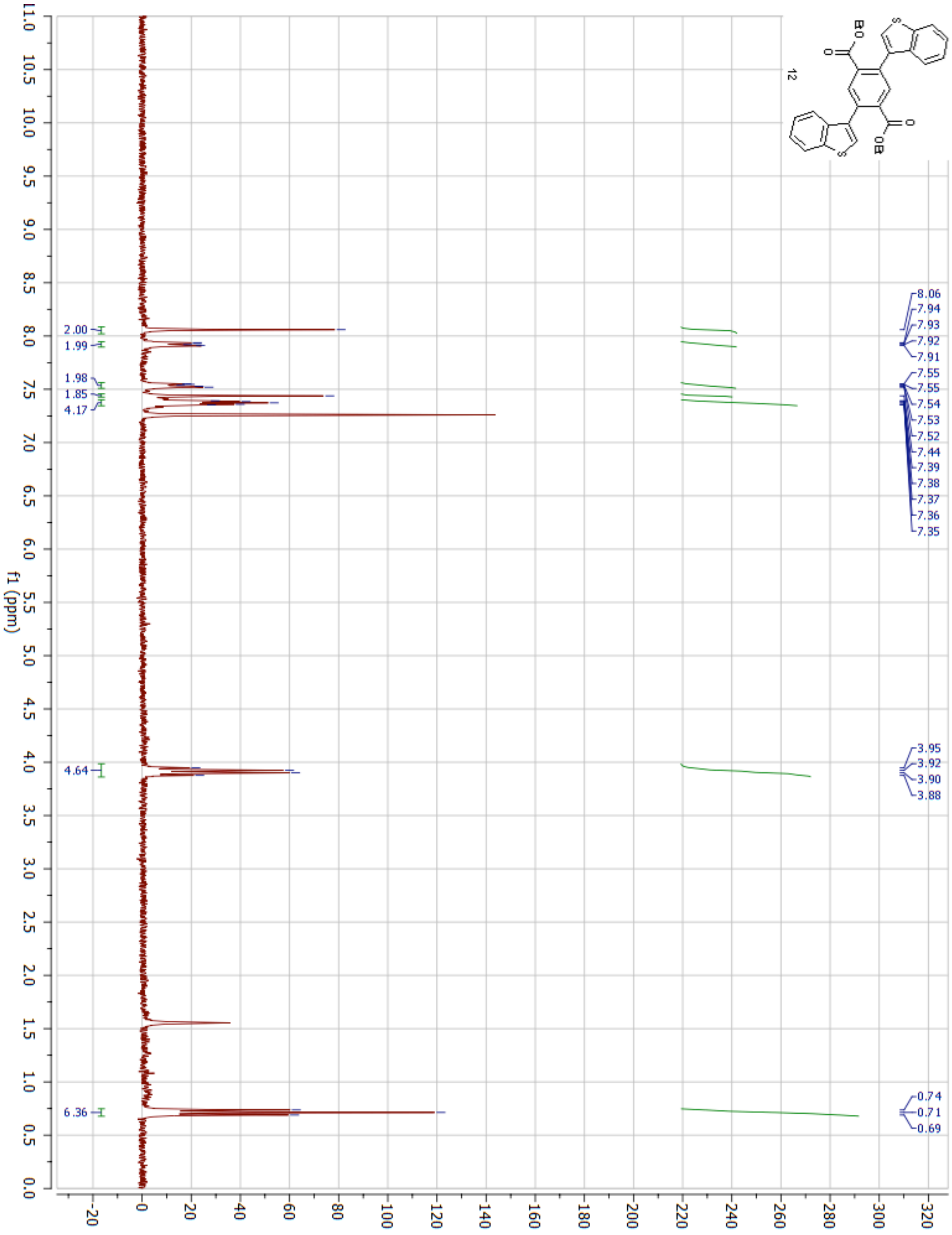
12
OEt
O
S
8.06
7.94
7.93
7.92
7.91
7.55
7.55
7.54
7.53
7.52
7.44
7.39
7.38
7.37
7.36
7.35
3.95
3.92
3.90
3.88
0.74
0.71
0.69
2.00
1.99
1.98
1.85
4.17
4.64
6.36
f1 (ppm)

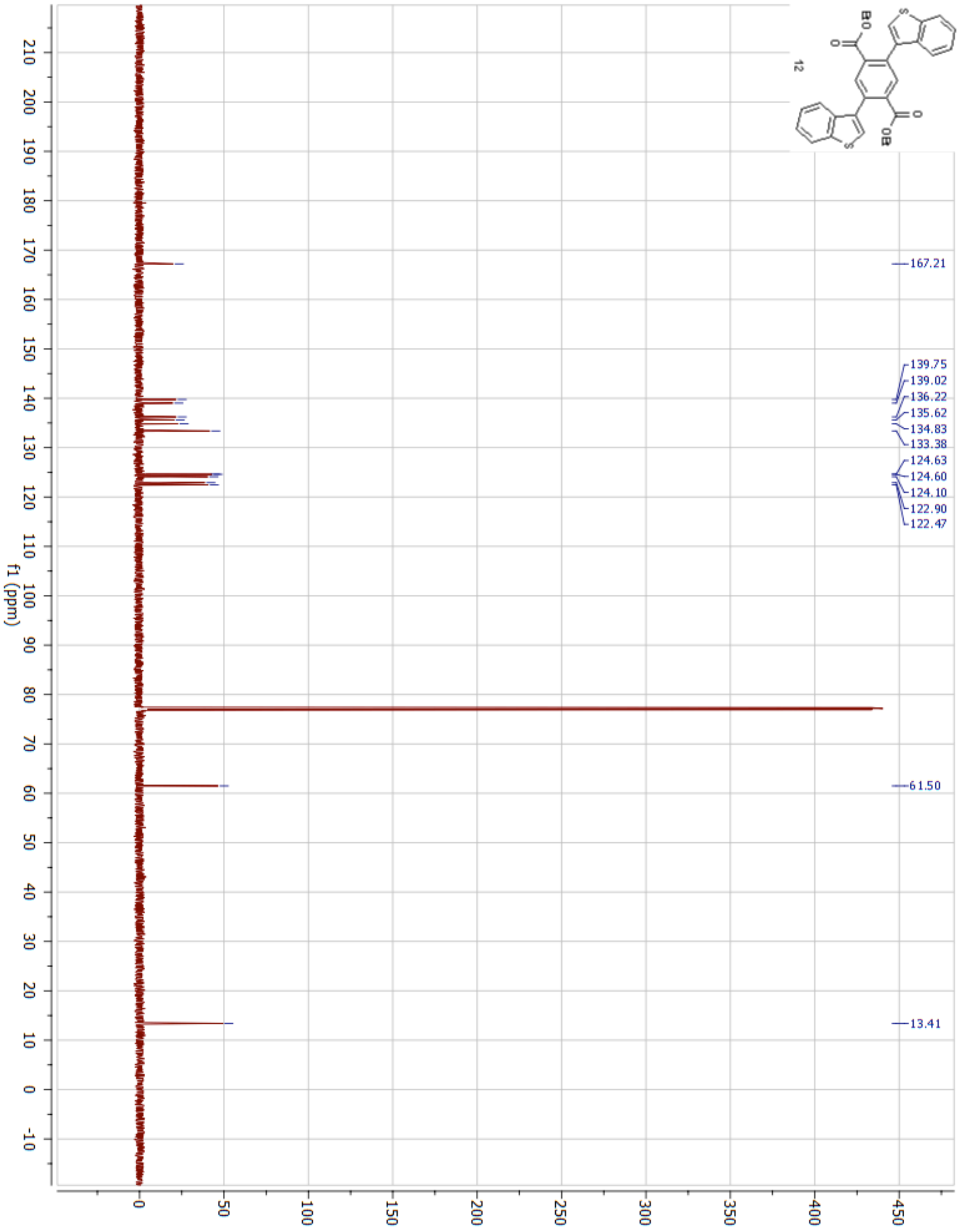
12
167.21
139.75
139.02
136.22
135.62
134.83
133.38
124.63
124.60
124.10
122.90
122.47
61.50
13.41
f1 (ppm)

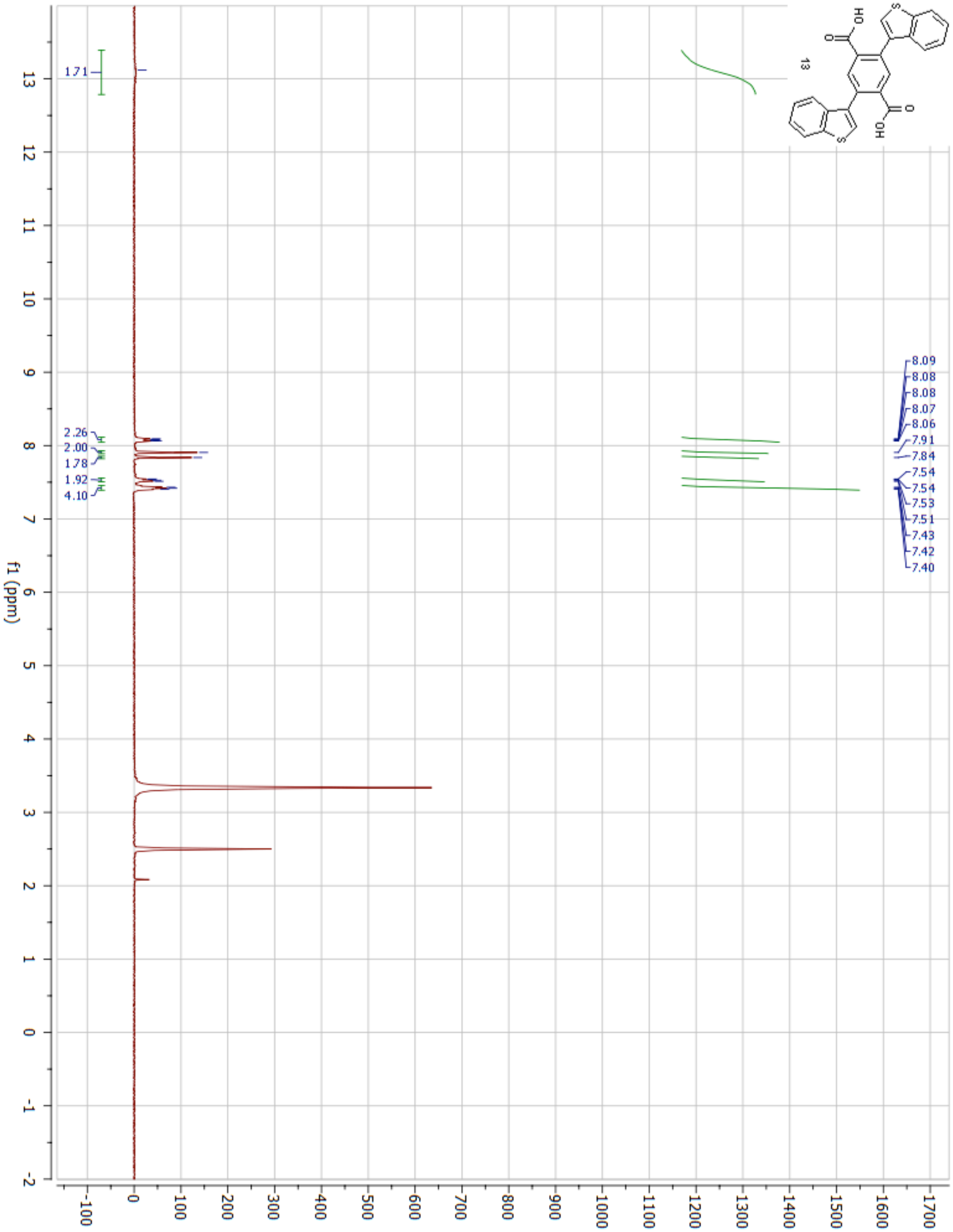
13
f1 (ppm)
1.71
2.26
2.00
1.78
1.92
4.10
8.09
8.08
8.08
8.07
8.06
7.91
7.84
7.54
7.54
7.53
7.51
7.43
7.42
7.40

Electronic Supplementary Material (ESI) for Chemical Science
This journal is © The Royal Society of Chemistry 2013

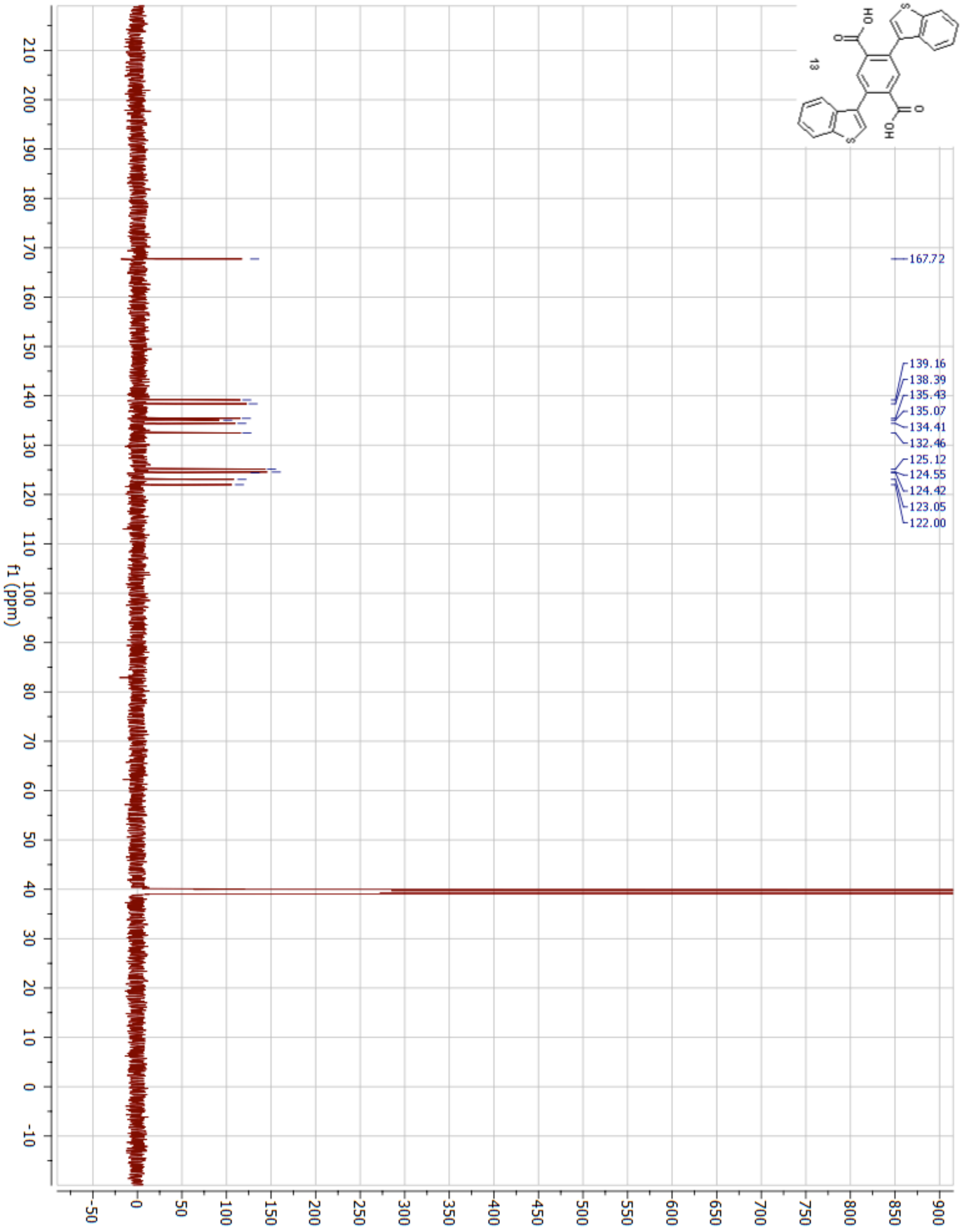

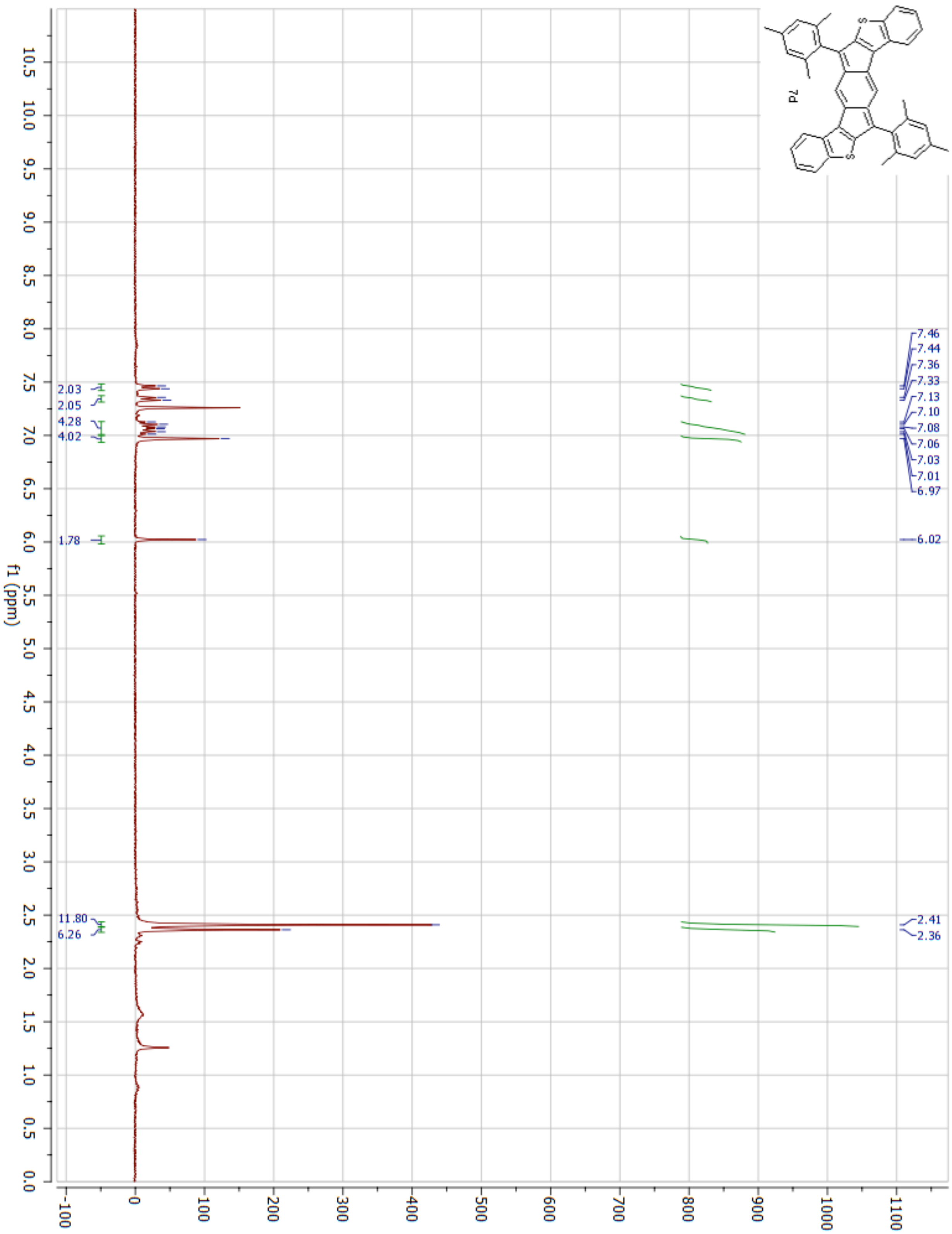
7d
f1 (ppm)
7.46
7.44
7.36
7.33
7.13
7.10
7.08
7.06
7.03
7.01
6.97
6.02
2.41
2.36
2.03
2.05
4.28
4.02
1.78
11.80
6.26

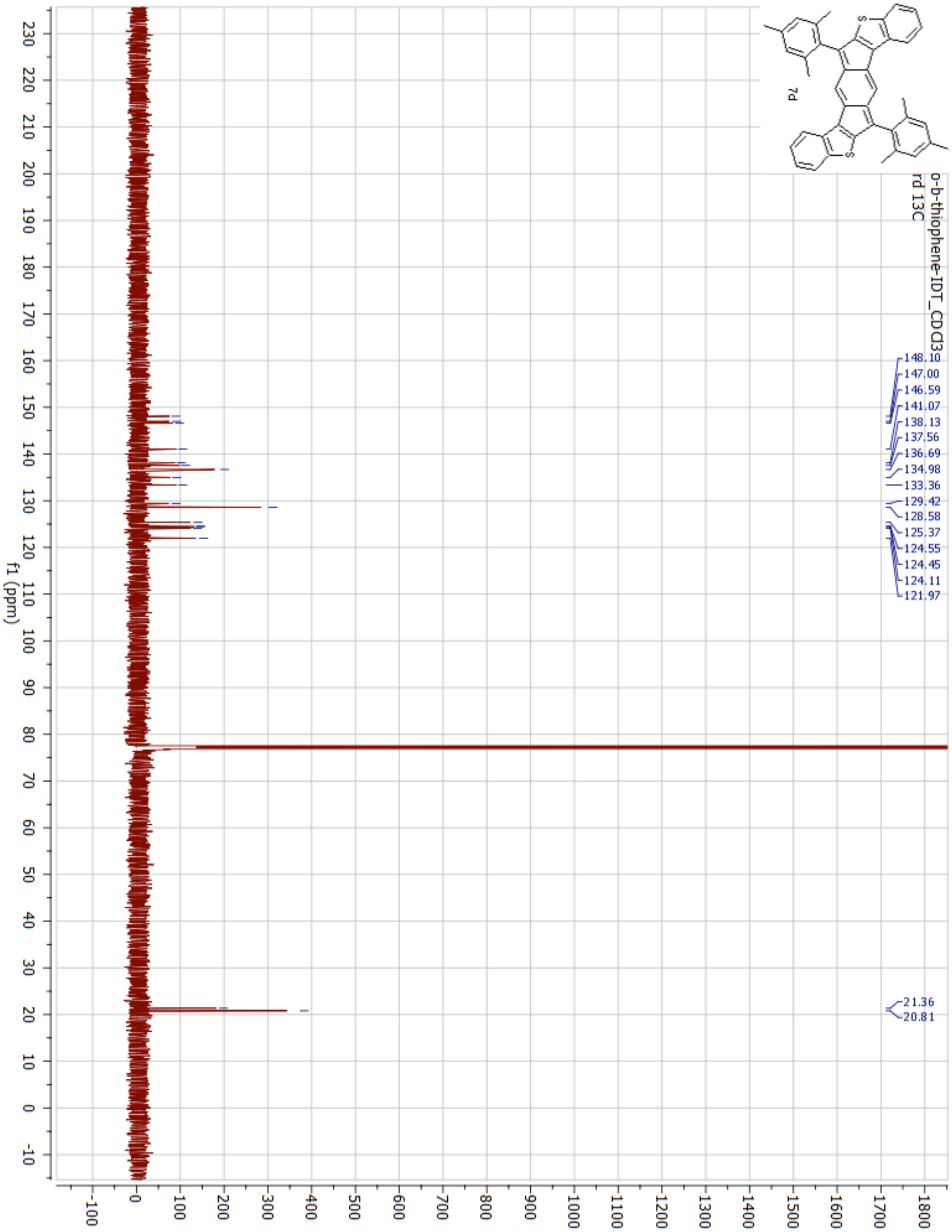
o-b-thiophene-IDT_CDCl3
rd 13C
7d
148.10
147.00
146.59
141.07
138.13
137.56
136.69
134.98
133.36
129.42
128.58
125.37
124.55
124.45
124.11
121.97
21.36
20.81
f1 (ppm)
